# The TINCR ubiquitin-like microprotein is a tumor suppressor in squamous cell carcinoma

**DOI:** 10.1038/s41467-023-36713-8

**Published:** 2023-03-10

**Authors:** Lucia Morgado-Palacin, Jessie A. Brown, Thomas F. Martinez, Juana M. Garcia-Pedrero, Farhad Forouhar, S. Aidan Quinn, Clara Reglero, Joan Vaughan, Yasamin Hajy Heydary, Cynthia Donaldson, Sandra Rodriguez-Perales, Eva Allonca, Rocio Granda-Diaz, Agustin F. Fernandez, Mario F. Fraga, Arianna L. Kim, Jorge Santos-Juanes, David M. Owens, Juan P. Rodrigo, Alan Saghatelian, Adolfo A. Ferrando

**Affiliations:** 1 Institute for Cancer Genetics, Columbia University, New York, NY USA; 2 Department of Pharmaceutical Sciences, University of California, Irvine, CA USA; 3 Department of Otolaryngology, Hospital Universitario Central de Asturias (HUCA), Oviedo, Spain; 4 Instituto de Investigación Sanitaria del Principado de Asturias (ISPA), Oviedo, Spain; 5 Instituto Universitario de Oncología del Principado de Asturias (IUOPA), Universidad de Oviedo, Oviedo, Spain; 6 Ciber de Cáncer, CIBERONC, Madrid, Spain; 7 Proteomics and Macromolecular Crystallography Shared Resource, Herbert Irving Comprehensive Cancer Center, Columbia University, New York, NY USA; 8 Clayton Foundation Laboratories for Peptide Biology, Salk Institute for Biological Studies, La Jolla, CA USA; 9 Molecular Cytogenetics Group, Human Cancer Genetics Program, Centro Nacional de Investigaciones Oncológicas (CNIO), 28029 Madrid, Spain; 10 Cancer Epigenetics and Nanomedicine Laboratory, Nanomaterials and Nanotechnology Research Center (CINN-CSIC), El Entrego, Spain; 11 Department of Organisms and Systems Biology (B.O.S.), University of Oviedo, Oviedo, Spain; 12 Rare Diseases CIBER (ciberer) of the Carlos III Health Institute (ISCIII), Madrid, Spain; 13 Department of Dermatology, Columbia University Irving Medical Center, New York, NY USA; 14 Department of Dermatology, Hospital Universitario Central de Asturias (HUCA), Oviedo, Asturias Spain; 15 Dermatology Area, University of Oviedo Medical School, Oviedo, Asturias Spain; 16 Department of Pathology and Cell Biology, Columbia University Irving Medical Center, New York, NY USA; 17 Department of Systems Biology, Columbia University, New York, NY USA

**Keywords:** Head and neck cancer, Head and neck cancer, Squamous cell carcinoma, Tumour-suppressor proteins

## Abstract

The *TINCR* (Terminal differentiation-Induced Non-Coding RNA) gene is selectively expressed in epithelium tissues and is involved in the control of human epidermal differentiation and wound healing. Despite its initial report as a long non-coding RNA, the *TINCR* locus codes for a highly conserved ubiquitin-like microprotein associated with keratinocyte differentiation. Here we report the identification of TINCR as a tumor suppressor in squamous cell carcinoma (SCC). TINCR is upregulated by UV-induced DNA damage in a TP53-dependent manner in human keratinocytes. Decreased TINCR protein expression is prevalently found in skin and head and neck squamous cell tumors and TINCR expression suppresses the growth of SCC cells in vitro and in vivo. Consistently, *Tincr* knockout mice show accelerated tumor development following UVB skin carcinogenesis and increased penetrance of invasive SCCs. Finally, genetic analyses identify loss-of-function mutations and deletions encompassing the *TINCR* gene in SCC clinical samples supporting a tumor suppressor role in human cancer. Altogether, these results demonstrate a role for *TINCR* as protein coding tumor suppressor gene recurrently lost in squamous cell carcinomas.

## Abstract (web summary)

TINCR encodes a p53-regulated ubiquitin-like microprotein expressed in stratified epithelia. Tincr loss promotes UVB-induced skin carcinogenesis in mice and deletions and mutations in human squamous cell carcinoma support a tumor suppressor role.

## Introduction

The discovery of transcriptional units without apparent protein-coding activity in the genome, followed by the functional characterization of many of these long non-coding RNAs (lncRNAs) as important factors involved in chromatin remodeling and in transcriptional and post-transcriptional regulation of gene expression, has brought to the forefront an essential role for these non-coding elements in shaping the architecture of transcriptional networks controlling cell and tissue homeostasis and disease^1,2^. Disruption of the homeostatic mechanisms controlling epidermal cell proliferation and differentiation is of relevance to the pathogenesis of squamous cell carcinoma, a highly prevalent cancer worldwide, and numerous lncRNAs regulate epidermal development and differentiation^3^, pointing to a role of these transcriptional units in the pathogenesis of human cancer. Among these, the *TINCR* gene is prominently expressed in human skin and has a proposed non-coding RNA-mediated role in the control of epidermal cell differentiation^4^. Moreover, deregulated *TINCR* expression has been observed across several different epithelial tumor types^5^. However, a potential protein-coding role for *TINCR* has been proposed based on global proteomic profiling of cornified epidermal keratinocytes, questioning the lncRNA nature of the *TINCR* transcript and recent reports have documented and characterized the TINCR locus as a protein-coding gene^6–8^. Here, we document the activation of *TINCR* expression downstream of P53 activation following UV-induced DNA damage, show increased incidence of SCC in *Tincr* knockout mice following UV-induced carcinogenesis and describe the presence of loss of function protein-truncating mutations and deletions encompassing the *TINCR* locus, all in support of a tumor suppressor role in human SCC. Moreover, we demonstrate that expression of the TINCR protein can impair tumor growth in squamous cell carcinoma cell lines and document that loss of TINCR protein expression is associated with poor prognosis in metastatic cutaneous SCC.

## Results

To explore the potential role of TINCR in tissue homeostasis and diseases we first characterized its expression across tissues. In agreement with previous reports^4,7,8^, we detected expression of TINCR transcripts in stratified epithelial tissues including the esophagus, trachea, and cervix. *TINCR* expression is particularly prominent in the skin (Supplementary Fig. 1a–c) and the TINCR protein is readily detected in cutaneous basal keratinocytes with a prominent upregulation in more differentiated spinocellular and granular skin layers (Supplementary Fig. 1d). In addition, and consistent with the proposed role of *TINCR* RNA in terminal keratinocyte differentiation^4,7,8^, the *TINCR* messenger RNA and protein are markedly upregulated upon in vitro calcium-induced differentiation of primary human keratinocytes (Supplementary Fig. 1e–g).

**Fig. 1:**
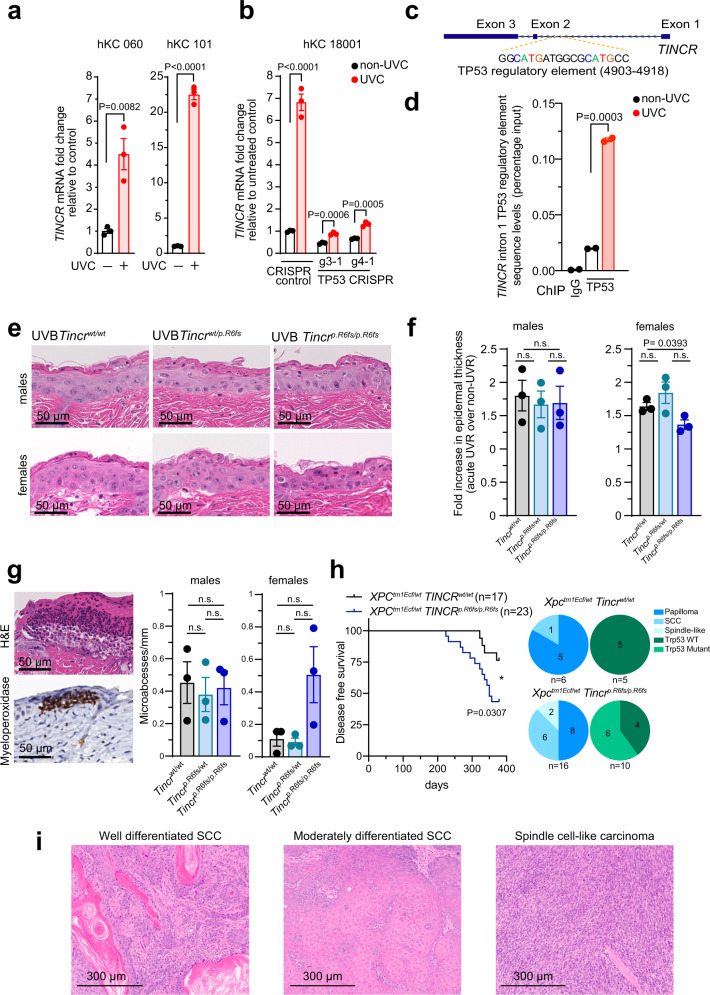
*TINCR* is a p53 target gene upregulated in response to UV-induced damage. **a** RT-PCR of *TINCR* levels in human keratinocytes from two foreskin samples (060 and 101) at baseline and following UVC radiation (100 mJ/cm^2^) as average values normalized to *ACTB* relative to untreated controls. Error bars: standard error of the mean in technical replicates. *P* values: two-tailed unpaired Student’s *t*-test. **b** *TINCR* RNA levels in wild type and CRISPR *TP53* knockout human keratinocytes targeted by gRNAs (g3-1, g4-1) in basal conditions and 4 h following UVC radiation (20 mJ/cm^2^) as in **a**. **c** Localization of the intron 1 TP53 regulatory element in the *TINCR* locus. **d** Quantitative PCR analysis of TP53 antibody chromatin immunoprecipitation with primers flanking the TP53 regulatory element in human keratinocytes at baseline and following UVC treatment. IgG chromatin immunoprecipitation is shown as control. *P* values: two-tailed unpaired Student’s *t*-test. **e** Representative images of hematoxylin-eosin-stained back skin from UVB-treated wild type, heterozygous, and homozygous *Tincr* mutant mice. **f** Quantification of epidermal thickness following UVB radiation in male (*n* = 3) and female (*n* = 3) mice for each *Tincr* genotype as in **e** as average values of relative skin thickness normalized to non-UVB-treated controls for each sex and genotype. Error bars: standard error of the mean. *P* values: two-tailed unpaired Student’s *t*-test. **g** Hematoxylin-eosin and myeloperoxidase stains showing UVB-induced skin microabscesses, and quantification of the number of these lesions in males and females for each *Tincr* genotype. Graphs show average values, each dot represents one mouse, and error bars correspond to the standard error of the mean. *P* values: two-tailed unpaired Student’s *t*-test. **h** Kaplan–Meier disease-free survival curves of *Xpc*^tm1Ecf/wt^ *Tincr*^wt/wt^ (*n* = 17) and *Xpc*^tm1Ecf/wt^ *Tincr*^p.R6fs/p.R6fs^ (*n* = 23) mice following long-term exposure to UVB (100 mJ/cm^2^, 3 times per week for 35 weeks). *P* values: log-rank Mantel–Cox test. Pie chart graphs show numbers of papillomas (dark blue), squamous cell carcinomas (medium blue) or spindle cell-like carcinomas (light blue) and if lesions were *Trp53* wild-type (dark green) or *Trp53* mutant (light green) in *Xpc*^tm1Ecf/wt^ *Tincr*^wt/wt^ and *Xpc*^tm1Ecf/wt^ *Tincr*^p.R6fs/p.R6fs^ mice. **i** Haematoxylin-eosin-stained micrographs of SCC tumors developing following chronic UVB exposure in *Xpc*^tm1Ec/wt^ *Tincr*^p.R6fs/p.R6fs^ mice. Source data are provided as a Source Data file.

The barrier function of the skin epidermis plays an important protective role against dehydration, infections, and erosion that is challenged by DNA damage from daily sunlight exposure^9,10^. To explore a potential role for TINCR in the skin response to UV-induced damage we analyzed *TINCR* mRNA levels in human keratinocytes after UVC (100–280 nm) radiation. These analyses revealed marked transcriptional upregulation of *TINCR* transcripts upon UVC insult (Fig. 1a), suggesting a functional involvement downstream of the P53-mediated DNA damage response.

Consistent with this hypothesis, CRISPR knockout of *TP53* in human keratinocytes abrogated *TINCR* mRNA induction by UVC radiation (Fig. 1b). In agreement, transcription factor binding motif analyses identified a canonical P53 response element in the first intron of the *TINCR* gene (Fig. 1c). Moreover, P53 chromatin immunoprecipitation revealed enrichment of sequences encompassing this response element in UVC-treated primary human keratinocytes compared to non-UV-treated controls (Fig. 1d). These results were specific to UV-induced DNA damage, as a genotoxic insult with the alkylating agent cisplatin and the antimetabolite hydroxyurea, did not increase *TINCR* expression, potentially reflecting limited induction of DNA damage by these agents in non-actively proliferating primary human keratinocytes (Supplementary Fig. 1h). In all, these results characterize *TINCR* as a direct target of P53 and upregulated in response to UV-induced DNA damage.

UV irradiation-induced damage in the mouse skin triggers an inflammatory response with neutrophil aggregation, epidermal hyperplasia, and desquamation of the terminally differentiated skin layers. To test the functional role of TINCR in response to UV exposure in vivo, we genetically engineered mice harboring a frameshift mutation (c.15_16insT) that proximally alters the mouse *Tincr* reading frame (p.Arg6Thrfs*33) with minimal alteration of the secondary structure of the *Tincr* lncRNA (Supplementary Fig. 2a, b). Of note, no alternative downstream translation initiation codons exist in the Tincr canonical open reading frame and the predicted polypeptide encoded by the p.Arg6Thrfs*33 mutant allele (MEELRSRAVPLEALPHQGTPSRRGIATATDRAAPRHTE*) shows no similarity with the Tincr protein after the first 5 amino acids. *Tincr* mutant mice are viable and fertile with Mendelian segregation of the mutant allele. Given that male and female mice show differential skin structural features and response to UV^11,12^, we analysed separately the skin phenotypes of *Tincr* wild type and mutant male and female mice. These analyses revealed preserved epithelial integrity and no differences in epithelial thickness, architecture, or keratinocyte differentiation in *Tincr* wild-type and mutant animals in homeostatic conditions (Supplementary Fig. 3a–c). However, treatment of *Tincr* wild type, heterozygous and knockout mice with an erythema-inducing dose of UVB (280–315 nm) radiation revealed a significant reduction (*P* = 0.0393) of the UVB-induced epidermal thickening at 48 h in *Tincr* knockout females compared to wild type controls (Fig. 1e, f). In addition, *Tincr* knockout female mice showed a higher number of UVB-induced neutrophil microabscesses compared to their male counterparts (Fig. 1g).

Impaired resolution of inflammatory responses following UV-induced injury in the skin of *Tincr* deficient mice, together with our observation of *TINCR* upregulation by P53 following UV-induced genotoxic stress, suggested a potential tumor suppressor role for *TINCR* in the skin and stratified epithelia. To test the role of *TINCR* as a tumor suppressor, we performed a UVB skin carcinogenesis experiment in the UV-induced DNA damage repair defective *Xpc* heterozygous mutant background, which sensitizes mice to UVB carcinogenesis in the skin^13^. In these experiments, we treated *Tincr* wild type, *Xpc* heterozygous (*Xpc*^*tm1Ecf/wt*^ *Tincr*^*wt/wt*^) and *Tincr* mutant, *Xpc* heterozygous (*Xpc*^*tm1Ecf/wt*^ *Tincr*^*p.R6fs/p.R6fs*^) mice with UVB (100 mJ/cm^2^) three times a week for 35 weeks. In this experiment, we observed accelerated papilloma skin lesions on the UVB-exposed back skin of *Tincr* mutant mice (32-week latency) compared with *Tincr* wild-type controls (46-week latency) (Fig. 1h). In total, 13 mice developed papillomas and squamous cell carcinomas in *Xpc*^*tm1Ecf/wt*^ *Tincr*^*p.R6fs/p.R6fs*^ mice, while only 4 *Xpc*^*tm1Ecf/wt*^ *Tincr*^*wt/wt*^ mice developed skin lesions by the experimental endpoint of 54 weeks (*P* = 0.0307) (Fig. 1h and Supplementary Data 1). We also observed increased progression to carcinoma and specific development of invasive tumors with spindle-like features in UV-treated *Tincr* mutant mice (Fig. 1h, i and Supplementary Data 1). In addition, only tumors from *Tincr* deficient *Xpc*^*tm1Ecf/wt*^ *Tincr*^*p.R6fs/p.R6fs*^ mice gained *Trp53* mutations over the course of these UVB carcinogenesis experiments (Fisher’s exact test *P* = 0.04) (Fig. 1h and Supplementary Data 2).

**Fig. 2:**
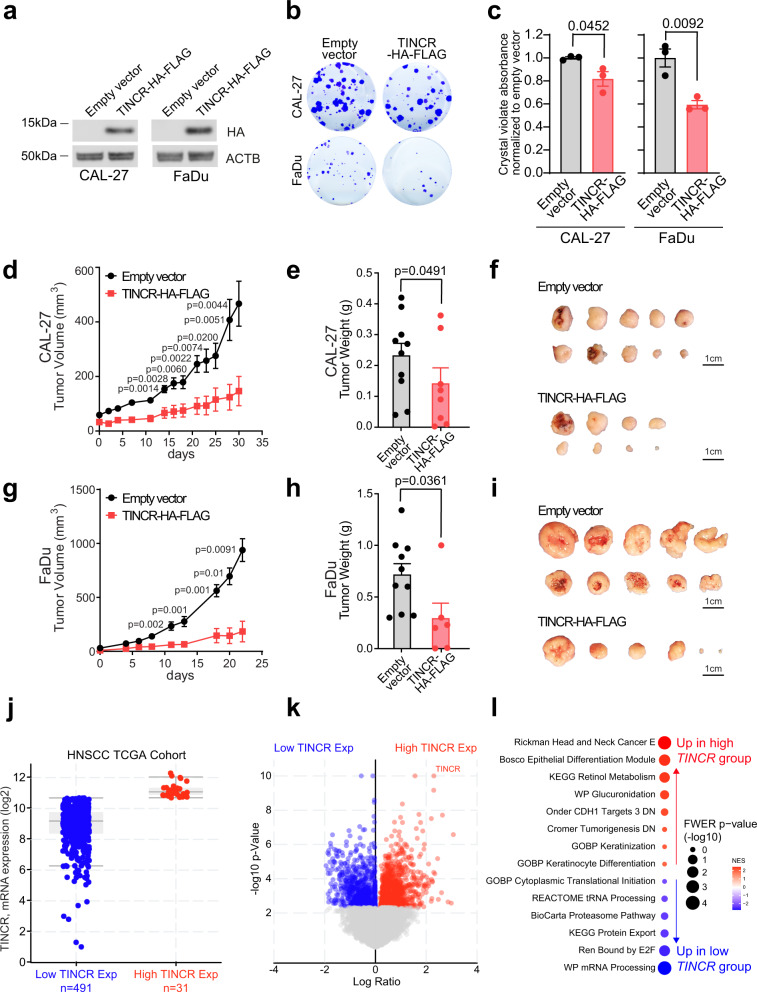
TINCR suppresses squamous cell carcinoma cells and tumor growth. **a** Western blot analysis of exogenous TINCR protein expression in HNSCC cell lines infected with empty or TINCR-HA-FLAG expression vectors. **b** Representative images of colony-forming assays in CAL-27 and FaDu cells expressing either empty vector or TINCR-HA-FLAG constructs. **c** Quantification of colony growth as in **b**. Graphs show average crystal violet signal across technical triplicates. Error bars show the standard error of the mean. Data were representative of two independent experiments. **d** In vivo intradermal tumor growth of CAL-27 cells expressing either empty vector or TINCR-HA-FLAG constructs. Growth curves show the average volume and standard deviation across ten independent tumors for each condition. Error bars show the standard error of the mean. **e** Graph shows the average weight of tumors recovered at the endpoint for each condition as in **d**. Error bars show the standard error of the mean. **f** Images of tumors recovered at endpoint for each condition as in **e. g** In vivo intradermal tumor growth of FaDu cells expressing either empty vector or TINCR-HA-FLAG constructs. Growth curves show the average volume and standard deviation across ten independent tumors for each condition. Error bars show the standard error of the mean. **h** Graph shows the average weight of tumors recovered at the endpoint for each condition as in **g**. Error bars show the standard error of the mean. **i** Images of tumors recovered at endpoint for each condition as in **g. j** Box and whisker plot of *TINCR* mRNA expression across TCGA HNSCC patients with high (*n* = 31, red) and low (*n* = 491, blue) *TINCR* expression. **k** Volcano plot representation of differential gene expression between HNSCC patients with high and low expression of *TINCR*. **l** Dot plot representation of GSEA analysis in HNSCC patients with high *TINCR* expression compared to HNSCC patients with low *TINCR* expression. *P* values in **c**–**e**, **g**, **h** correspond to two-tailed unpaired Student’s *t*-test. Source data are provided as a Source Data file.

To further explore the tumor suppressor role of TINCR in human SCCs, we evaluated the impact of mimicking P53-induced TINCR upregulation by lentivirally expressing the TINCR protein-coding sequence in *TP53* mutant CAL-27 (*TP53* homozygous c.578 A > T) and FaDu (*TP53* c.376-1 G > A, c.743 G > T) head and neck squamous carcinoma (HNSCC) cell lines, which lack the expression of endogenous TINCR (Supplementary Fig. 3d, e). These experiments revealed reduced numbers of colony-forming units, slightly limited colony size, decreased cell growth, and delayed proliferation in cells ectopically expressing TINCR protein compared with controls (Fig. 2a–c). Moreover, TINCR protein expression impaired the growth and tumorigenicity of CAL-27 (Fig. 2d–f) and FaDu (Fig. 2g–i) cell xenografts in vivo. Moreover, and in support of a differentiation role for TINCR as a tumor suppressor, gene expression analysis of HNSCC patient samples from TCGA^14^ revealed an association of *TINCR* RNA levels with transcriptional programs linked with epithelial differentiation and keratinization (Fig. 2j–l and Supplementary Data 3, 4,5).

**Fig. 3:**
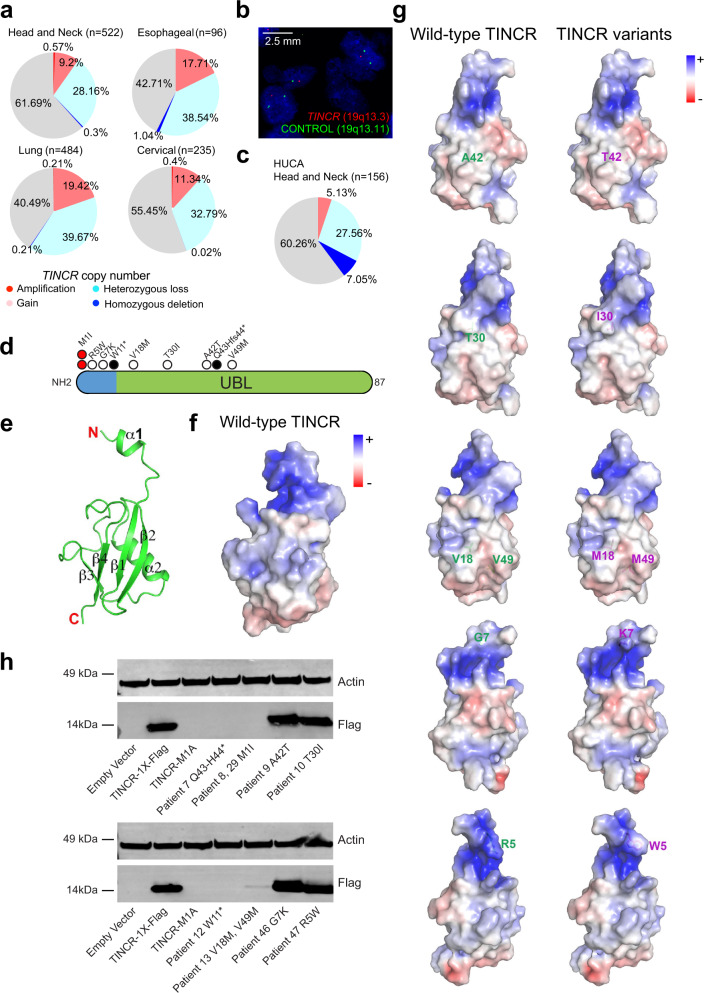
Prevalence and structural consequence of TINCR deletions in human cancer. **a** Frequency of *TINCR* copy number alterations in TCGA HNSCC, lung SCC, esophageal SCC, and cervical SCC tumors. **b** Representative image of *TINCR* heterozygous loss detection by fluorescence in situ hybridization (FISH) in HNSCC. **c** Pie chart representation of frequency TINCR copy number alterations in TCGA HNSCC analyzed by FISH. **d** Schematic representation of the TINCR protein indicating the position of mutations identified in HNSCC patient tumor samples. Translation-initiating codon mutations are depicted as red circles, truncating mutations as black circles, and single amino acid substitutions as white circles. **e** Ribbon diagram representation of TINCR protein crystal structure at 2.12 Å resolution. **f** Electrostatic surface potential of the wild-type TINCR protein. Positively charged regions are shown in blue (kT/e = +5) and negatively-charged regions are shown in red (kT/e = −5). **g** Electrostatic surface potential of the documented TINCR variants. Positively charged regions are shown in blue (kT/e = +5) and negatively-charged regions are shown in red (kT/e = −5). **h** Western blot of analysis of documented TINCR variants on TINCR protein expression in 293 cells infected with empty vector, TINCR-1X-Flag, or variant TINCR constructs. Source data are provided as a Source Data file.

Following these findings, we explored the presence of genetic alterations involving the *TINCR* locus in squamous cell carcinoma clinical samples. Genetically, copy number alteration analysis revealed highly prevalent heterozygous chromosomal losses encompassing the *TINCR* locus across head and neck (~28%), lung (~39%), esophageal (~38%), and cervical (~32%) squamous cell carcinomas in TCGA cohorts (Fig. 3a). Consistently, fluorescence in situ hybridization analysis of the *TINCR* gene showed ~27% heterozygous deletions and 7% homozygous loss in an independent series of oropharyngeal squamous cell carcinomas (*n* = 156) (Fig. 3b, c). In addition, dideoxynucleotide sequencing of DNA samples from HNSCC tissue biopsies revealed protein-altering mutations in 9/55 cases (Fig. 3d–g and Supplementary Fig. 4a–c). These included compound heterozygous mutations (p.Val18Met; p.Val49Met) in one tumor sample and homozygous mutations in five additional cases (Fig. 3g and Supplementary Fig. 4b). In two cases a recurrent single nucleotide point mutation (p.Met1Ile) altered the translation initiation codon and two cases showed early truncating mutations (p.Trp11* and p.Gln43Hisfs44*).

To best evaluate the impact of these alterations we analysed the structure of the TINCR protein. Structural characterization of TINCR at 2.12 Å resolution revealed a UBL fold domain composed of four β-strands forming one β-sheet (β1-4) and one α-helix (α2) (Fig. 3e), the lack of a characteristic C-terminal di-Glycine motif required for covalent protein conjugation that is present in all other ubiquitin-like proteins described so far with the exception of UBL5^15^ (Fig. 3e and Supplementary Fig. 4d–f), and a 15-residue long highly conserved positively charged N-terminal extension (herein referred to as “cap”) as the most prominent features (Supplementary Fig. 4d–f and Supplementary Data 6, 7). The “cap” is visible only in protomer A, as it is buttressed by two neighboring TINCR protomers. However, its high B-factor values suggest that it is highly mobile in solution. Projection of *TINCR* amino acid variants identified in SCC samples on this structure revealed a protein disruptive effect of these non-conservative amino acid substitutions (p.Arg5Trp; p.Gly7Lys; p.Val18Met; p.Thr30Ile; p.Ala42Thr; p.Val49Met) (Fig. 3d–g). The distribution of these point mutations across both the N-terminal cap region and the ubiquitin-like domain, together with recurrent loss of translation-initiating codon mutations, N-terminal proximal truncating lesions, and locus deletions, all support a loss-of-function mechanism. To confirm the damaging effects of these mutations on TINCR protein expression, we generated expression constructs for each of the TINCR variants detected in HNSCC patients. Western blot analysis of TINCR protein in cells transfected with these vectors revealed that, as expected, loss of TINCR protein products from cells transfected with initiating codon altering and truncating mutations (Fig. 3h). Similarly, the introduction of a compound p.V18M, p.V49M mutation resulted in almost complete abrogation of TINCR protein expression (Fig. 3h). Albeit, in-depth functional characterization of the predicted deleterious alleles with retained TINCR expression remains to be addressed, these results argue for a loss of function mechanism.

**Fig. 4:**
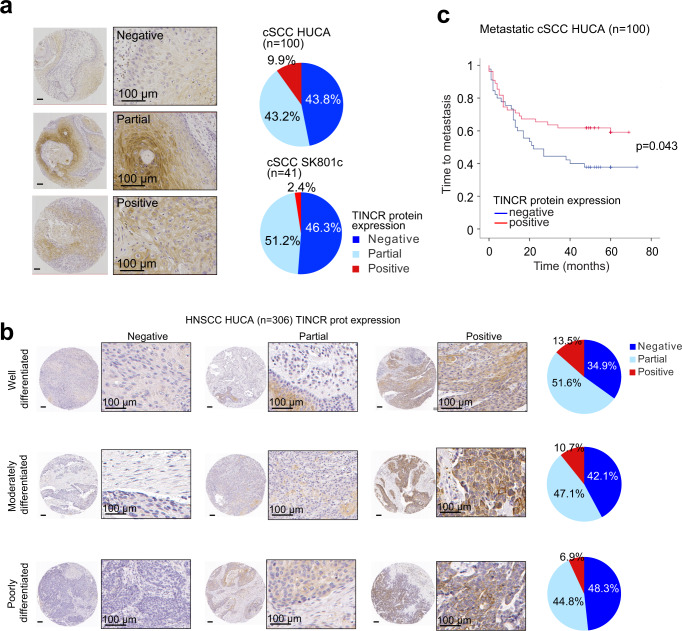
Prognostic impact of the loss of TINCR protein expression in human cancer. **a** Representative images of immunohistochemical analysis of TINCR protein expression in cSCC tumors. Pie chart graphs show the prevalence of different TINCR protein expression patterns in two independent cohorts of cSCC samples. Negative (blue): no detection. Partial (light blue): TINCR expression is limited to differentiated areas of the tumor. Positive (red): diffuse TINCR staining. **b** Representative images of the different staining patterns of TINCR detected by immunohistochemistry in HNSCC patient tumor samples classified according to their differentiation grade. Pie chart graphs depict the distribution of TINCR protein expression patterns in relation to the tumor differentiation grading in a cohort of 306 HNSCC cases. **c** Kaplan–Meier curves indicating time to metastasis in patients with metastatic cSCC dichotomized according to TINCR protein expression.

To further explore the impact of TINCR inactivation in human cancer we performed immunohistochemical analysis across two independent cohorts of cutaneous squamous cell carcinoma (cSCC) samples encompassing a total of 141 patient samples (HUCA *n* = 100; cSCC SK801c *n* = 41). Across these series, we observed loss of TINCR protein expression in over 40% of these cases (Fig. 4a, Supplementary Fig. 5, and Supplementary Data 8,9). Similarly, loss of TINCR protein was detected in over 30% of patient samples from an HNSCC cohort (HUCA *n* = 306) across all tumor differentiation grades. Of note, the most aggressive poorly differentiated tumors presented the highest frequency of negative TINCR expression (48%) in this series, though TINCR expression did not associate with survival (Fig. 4b and Supplementary Fig. 5). TINCR expression in HNSCC showed a trend in positive correlation with CDKN1A (p21) levels, a readout of TP53 activity (Supplementary Data 10). In addition, lymph node metastasis and a tumor thickness of less than 6 mm was correlated with negative expression of TINCR protein (Supplementary Data 10). Across our HNSCC series, HPV was detected in ten cases, five of which showed loss of TINCR expression. Moreover, evaluation of clinical outcomes in a selected cohort of cSCC with nodal metastasis further revealed that patients with TINCR-negative tumors exhibited more frequent metastasis (log-rank test, *p* = 0.043) and a trend towards lower overall survival (log-rank test, *p* = 0.089) compared with those harboring either diffuse positive TINCR protein expression or partial expression in differentiated areas of the tumor (Fig. 4c, Supplementary Fig. 5, and Supplementary Data 11), a result that is in line with the tumor suppressive effects of the TINCR protein observed in our UVB skin carcinogenesis and SCC xenograft experiments.

## Discussion

The identification of a highly conserved ubiquitin-like peptide encoded in the *TINCR* gene^6–8^, originally described as a long non-coding RNA locus selectively expressed in squamous epithelial tissues^4^, has prompted renewed interest in the functional characterization of this locus in tissue homeostasis and disease. Ubiquitin-like proteins often serve as substrates for protein conjugation and function as signal tags that are involved in protein degradation, subcellular localization, signal transduction, and epigenetic regulation^16^. However, unlike most ubiquitin-like factors, the TINCR protein lacks a characteristic C-terminal di-glycine motif^7^ (valine glycine motif in UFM1) required for protein conjugation^16^. The absence of this sequence in TINCR points to a specific role and suggests that this ubiquitin-like factor may not function as a substrate for lysine protein conjugation. Still, annotation of TINCR-associated protein complexes has suggested a potential role in the regulation of the proteasome^7^. Finally, the TINCR crystal structure stands out for the presence of a distinct basic N-terminal “cap”, whose highly positive charge suggests a potential role as a recognition module for either phospholipids, negatively-charged regions of proteins, or nucleic acids.

The *TINCR* mRNA and protein are readily detectable in the skin and, albeit at lower levels, in other stratified epithelial tissues, in line with the role of TINCR in the organization and function of the skin. Even though suppression of *TINCR* expression by siRNA knockdown can result in the impaired organization of three-dimensional human keratinocyte cultures in vitro^4^, *Tincr* knockout mice have been reported to have a mild skin phenotype primarily characterized by defects in skin wound healing^7^. However, we observed altered resolution of UV-induced skin damage (decreased epidermal thickening and increased neutrophil micro-abscess) in female *Tincr* knockout mice in support of a physiologic role of TINCR as a protective factor against UV light exposure. The presence of a more severe phenotype in UV-treated *TINCR* female knockout animals is not surprising given the prominent differences in skin thickness and response to injury between male and female mice^11,12^ and highlights the need to carefully consider sex as a biological variable in the characterization of phenotypes involving this tissue.

Importantly, a defect in the physiologic response to UV exposure potentially links TINCR with pathologies derived from excessive UV-induced skin damage including cancer. This possibility is further supported by the identification of *TINCR* as a direct P53 target gene upregulated following UV-induced genotoxic stress, particularly considering that the P53 tumor suppressor plays a major role as a guardian of genomic integrity in the skin, a tissue exposed daily to UV-induced DNA damage, and that mutational disruption of *TP53* is a common clonal genetic alteration in cSCC^17^. In agreement with this model, chronic UV exposure in *Tincr* knockout mice heterozygous for the *Xpc* DNA repair factor resulted in increased penetrance of skin tumors and progression to carcinoma. Moreover, squamous carcinoma cell lines show no detectable TINCR expression and lentiviral expression of the *TINCR* coding mRNA sequences resulted in impaired cell growth. Of note, the antiproliferative effect of TINCR re-expression was more prominently detected in vivo than in two-dimensional cultures in vitro and gene expression profiling analyses point to elevated cell adhesion and differentiation pathways as downstream effectors.

Discordant correlative studies analysing *TINCR* transcript levels in human cancer have associated decreased *TINCR* levels with colorectal^18^, prostate^19^, and non-small cell lung carcinomas^20,21^ and increased *TINCR* RNA expression in hepatocellular^22^, nasopharyngeal^23,24^, breast^25,26^, bladder^27^, gastric, and esophagus carcinomas^28–32^. However, a recent report published while this manuscript was under revision shows convergent results in support of TINCR regulation downstream of TP53 activation and linking TINCR activity with a potential tumor suppressor role in epithelial tissues^8^. This hypothesis is solidly substantiated here by our demonstration of common *TINCR* gene loss and the presence of recurrent protein-disrupting mutations in squamous cell carcinoma tumors. In addition, even though *TINCR* was originally described as a lncRNA^4,33,34^, the presence of protein-disrupting mutations including, and most telling, initiating codon-disrupting single nucleotide substitutions, together with the antiproliferative phenotype of TINCR protein expression in HNSCC tumor cells strongly support that the tumor suppressor function of the *TINCR* gene is mediated by the TINCR ubiquitin-like microprotein.

The observed deregulation of *TINCR* RNA in multiple tumor types and the association of *TINCR* expression with epidermal differentiation argue for an in-depth characterization of the transcriptional regulatory networks controlling *TINCR* expression beyond its role as a TP53 target gene. In particular, it will be important to explore the potential role of *TINCR* downstream of proliferation-driving oncogenic (RAS) and tissue organization and differentiation tumor suppressor (NOTCH) pathways commonly disrupted in squamous cell carcinoma. Finally, extended analysis of homogeneously treated and clinically annotated tumor cohorts will be instrumental in better defining the association of loss of TINCR protein expression with aggressive poorly differentiated tumor histopathology, metastatic disease, and outcomes.

## Methods

### Generation of mutant mice

We generated *Tincr* mutant mice at the Herbert Irving Comprehensive Cancer Center Transgenic Shared resource by cytoplasmic injection of fertilized eggs from C57BL/6 females with assembled RNP complexes, comprising recombinant Cas9 protein, synthetic sgRNA (5′-CCATGGAGGAGCTGCGGCGA-3′ with 2′-*O*-methyl 3′ phosphorothioate modifications in the first and last three nucleotides, Synthego) and a homologous recombination template of 100 nucleotides containing a mutant translation starting ATG codon (ssODN, SIGMA, PAGE-purified). To identify *Tincr (Gm20219*) mutant mice, we genotyped F1 founders by PCR amplification of genomic DNA using primers flanking the gRNA cleavage site (Forward: 5′-AAGCCATCACCATCCCACTG-3′, Reverse: 5′-TGACCTGCGAGAGAGTCTCA-3′) followed by PCR purification (QIAGEN) and Sanger DNA sequencing (Genewiz). No initiation codon mutations resulting from recombination with the mutant template were identified. A mutant line harboring a single nucleotide deletion creating an early frameshift protein-truncating allele (p.R6fs) was selected for analysis. We verified germline transmission of *Tincr* mutant alleles in F2 offspring and bred mice to homozygosity. *Tincr* homozygous mutant mice are fertile and viable with Mendelian segregation of the mutant allele. Heterozygous mutant mice for *Xpc* (B6; 129-*Xpc*^tm1Ecf^/J) were purchased from the Jackson Laboratory (Strain #010563)^13^. *Xpc* mutant mice were bred with *Tincr* mutant mice to generate mice heterozygous for *Xpc (Xpc*^tm1Ecf/wt^*)* and either wild-type (*Tincr*^wt/wt^) or homozygous mutant (*Tincr*^p.R6fs/p.R6fs^) for *Tincr*. All animals were maintained at the Irving Cancer Research Center at Columbia University Medical Campus in specific pathogen-free facilities. Animals were housed in a controlled environment at 20–24 °C temperature, 45–56% humidity, and 12-h light-dark cycles. Mice were fed a standard chow diet ad libitum. All animal procedures were evaluated and approved by the Columbia University Institutional Animal Care and Use Committee (IACUC).

### UV irradiation of mice

We used a UV Irradiation Unit (Daavlin Co., Bryan, OH) equipped with eight FS72T12-UVB-HO lamps that emit UVB (290-320 nm, 75–80% of total energy) and UVA (320–380 nm, 20–25% of total energy). The radiation emission is calibrated using an IL1700 Research Radiometer/Photometer (International Light Inc., Newburyport, MA) and the dose of UVB is quantified with a UVB Spectra 305 Dosimeter (Daavlin Co., Bryan, OH). The radiation source to the target distance is maintained at 38 cm, and no measurable increase in skin surface temperature occurs during the procedure. For acute UVB irradiation, we shaved mice with a hair clipper, exposed them on the following day to a single dose of 600 mJ/cm^2^, and collected skin samples for histological analyses 48 h after exposure. For long-term UVB skin carcinogenesis, we shaved mice once per week with a hair clipper and exposed them to a dose of 100 mJ/cm^2^ three times per week for 35 weeks. Mice were monitored for the number of tumors, tumor growth, and progression of papillomas to squamous cell carcinomas for 54 weeks.

### Cell cultures and treatment

We obtained HNSCC cell lines (CAL-27 #CRL-2095 tongue, FaDu #HTB-43 pharynx) from the ATCC repository. CAL-27 contains a homozygous c.578 A > T p.H193L mutation in TP53 and FaDu contains a heterozygous c.376-1 G > A p.? and heterozygous c.743 G > T p.R248L mutation in TP53. Cancer cells were grown in the following media supplemented with 10% FBS and antibiotics / antimycotics: DMEM (CAL-27), and MEM alpha Glutamax (FaDu). We obtained early passages of primary human keratinocytes (human keratinocytes) from neonatal foreskin samples under approved procedures by the Institutional Review Board. Primary mouse keratinocytes were obtained from the epidermis of neonatal pups on day 1 and subjected to dispase digestion (1 mg/mL) for 4 h at 37 °C. We cultured both primary human keratinocytes and mouse keratinocytes in a CnT-Prime medium without antibiotics/antimycotics (CnT-PR, CellnTec), and we used accutase (CnT-accutase-100, CellnTec) to detach cells when splitting was needed. Primary human keratinocytes and mouse keratinocytes were cultured for up to five passages. We irradiated human keratinocytes with UVC light at 20 or 100 mJ/cm^2^ in a Stratalinker 2400 equipped with five FG15T8 bulbs (254 nm wavelength) and let cells recover for 4 h or overnight, respectively. For primary human keratinocytes and mouse keratinocytes in vitro chemical-induced differentiation, we plated cells and CnT-PR media was replaced the following day by CnT-PR-D (2D differentiation) media containing 1.2 mM of CaCl_2_. We collected cells for further analyses at the indicated time points.

### Plasmids and vectors

The human *TINCR* full coding sequence encompassing 20 nucleotides upstream of the TINCR translation initiation codon and C-terminal HA-FLAG tag was synthesized by GenScript and cloned into the pCDH-CMV-MCS-EF1-puromycin lentiviral vector (Systems Biosciences CD510B). The mutant TINCR smORF sequences were synthesized using the BioXp system (Codex DNA). Mutant TINCR sequences were cloned into pcDNA 3.1+ expression vector linearized with KpnI/XbaI by Gibson assembly except for mutant sequence inserts from patients 7 and 12, which were cloned into the same vector but linearized with SnaBI/XbaI.

### CRISPR targeting of primary human keratinocytes

We used 293T-HEK cells to generate viral particles by transfecting with polyethylenimine (PEI) transfection reagent the corresponding lentiviral plasmids: lentiCRISPRv2-empty or lentiCRISPRv2 containing a single guide RNA against human *TINCR* (#116 5′-AGCCGGGCGGGCGCCATGGAGGG-3′ or #294 5′-CCTTCTACTACAACGCGCGGCGG-3′) and a single guide RNA against human p53 coding exons. We replaced the transfection media with fresh media on the following day and collected 48 h after transfection the lentiviral particles containing supernatant, which we filtered through a 45 µm PES filter before addition to the target cells. We infected primary human keratinocytes by spinoculation with the corresponding fresh raw viral supernatants and replaced viral media with fresh media after 3–4 h of recovery. We added puromycin (1 mg mL^−1^) on the following day and selected cells for 3 days.

### RNA isolation and quantitative real-time PCR (qRT-PCR)

We performed RNA extraction with the RNeasy Mini Kit (Qiagen, #74106) following the manufacturer’s instructions from cultured cells and mouse tissues collected and preserved in RNAlater (Millipore-SIGMA). Human total RNA samples were purchased from Thermo Fisher. RNA concentration and quality was measured with Nanodrop and equal amounts of RNA were converted into cDNA by using Superscript IV reverse transcriptase enzyme (Invitrogen). Real-time PCR was performed in a 7500 Real-Time PCR (Applied Biosystems) using TaqMan chemistry (Taqman master mix, Applied Biosystems) and TaqMan gene expression assays (Applied Biosystems, TINCR hS00542141_m1, and ACTB Hs00357333_g1). For quantification of the expression of the rest of the genes detected, SYBR chemistry was used (FAST-Start SYBR Green master mix, ROCHE) in combination with the corresponding primers indicated below. All reactions were performed in triplicates and normalized to *ACTB* mRNA levels as an endogenous control.

Human *KRT1* Fw 5′-TGAGCTGAATCGTGTGATCC-3′, Rv 5′-CCAGGTCATTCAGCTTGTTC-3′; human *FLG* Fw 5′-AAAGAGCTGAAGGAACTTCTGG-3′, Rv 5′-AACCATATCTGGGTCATCTGG-3′; human *LOR* Fw 5′-CTCTGTCTGCGGCTACTCTG-3′, Rv 5′-CACGAGGTCTGAGTGACCTG-3′; human *CDKN1A* Fw 5′-GATTAGCAGCGG AACAAGGAGT-3′, Rv 5′-TACAGTCTAGGTGGAGAAACGGG-3′; human *ACTB* F 5′-ACAGAGCCTCGCCTTTGC-3′, R 5′-AGGATGCCTCTCTTGCTCTG-3′; mouse *Klf4* Fw 5′-GTGCCCCGACTAACCGTT C-3′, Rv 5′- GTCGTTGAACTCCTCGGTCT-3′; mouse *Ivl* Fw 5′-GGGCAGAAACAGAAGCAGA-3′, Rv 5′-CAGTTCTGGCTCAGGTGACT-3′; mouse *Actb* Fw 5′-ACCTTCTACAATGAGCTGCG-3′, Rv 5′-CTGGATGGCTACGTACATGG-3′.

#### Gene expression profiling using the Cancer Genome Atlas Program

RNAseq transcriptional data of head and neck squamous cell carcinomas in The Cancer Genome Atlas (TCGA) database^14^ was queried in order to explore the transcriptional features of cases with high and low *TINCR* expression. We stratified tumor samples according to their *TINCR* expression score with a cutoff value of 1.5. Genes showing differential gene expression associated with high *TINCR* levels were extracted using cBioPortal. Next, Gene Set Enrichment Analysis (GSEA)^35,36^ was performed to define the Molecular Signatures Database (MSigDB)^37,38^ pathways associated with high and low TINCR expression in HNSCC patients. Genes and pathways associated with high and low *TINCR* expression are displayed in Supplementary Data 3, 4, 5.

### Generation of custom rabbit polyclonal antibody against TINCR protein

Custom rabbit polyclonal antibodies against TINCR protein were generated by immunization with a linearized KHL-conjugated peptide entailing an immunogenic conserved region between human and mouse that comprises the central region of TINCR from amino acids 30 to 44 at Covance and by immunization with an N-terminal (amino acids 2–25) TINCR peptide at the Salk Institute using standard procedures.

### Immunofluorescence

We carried out fluorescence detection of TINCR protein in frozen human foreskin sections that were blocked with 5% rabbit serum at room temperature for one hour and incubated with rabbit polyclonal anti-TINCR antibody (1:2000) for an additional 1 h at room temperature. We used an anti-rabbit AF488-conjugated secondary antibody raised in donkey (Invitrogen A21206) to detect fluorescence signal with a confocal microscope (Nikon Ti Eclipse inverted) and a 40X/1.19Oil or 60X/1.49Oil TIRF oil lens. DAPI was used to counterstain nuclei.

### Immunoblotting

Cells were harvested and lysed in RIPA buffer (50 mM Tris-Cl pH 8, 1 mM EDTA, 1% Triton-X100, 0.25% sodium deoxycholate, 0.1% SDS, 150 mM sodium chloride). Identical amounts of whole lysates were resolved on 4–12% SDS/PAGE gels (NuPAGE, Invitrogen) and transferred to nitrocellulose membranes. Blots were blocked in Odyssey Blocking buffer (LI-COR Odyssey) and incubated with the corresponding primary antibodies: rabbit monoclonal antibody anti-HA tag (CST #3724, clone C29F4, 1:1000 dilution), mouse monoclonal antibody anti-tubulin (SIGMA #T9026, clone DM1A, 1:5000 dilution), rabbit monoclonal antibody FLAG DYKDDDDK Tag (Cell Signaling, clone D6W5B, 1:1000 dilution) and mouse monoclonal antibody anti-β-Actin (SIGMA #5441, clone AC-15, 1:2000 dilution) and subsequently incubated with the corresponding secondary anti-IgG fluorescence-labeled antibodies (LI-COR Odyssey, 1:5000 dilution). Signals were acquired by an LI-COR Odyssey detector.

### Chromatin immunoprecipitation (ChIP) assay

ChIP assay was carried out as previously described in ref. ^39^, with some modifications. Briefly, we crosslinked control and UVC-treated primary hKCs, lysed cells, and sonicated lysates with the Bioruptor Pico device (Diagenode) at 8 °C for 30 cycles (30 s on, 30 s off). We quantified chromatin with the Qubit system (Thermo Fisher) and incubated 2 ug of crosslinked chromatin with 5 ug of normal rabbit IgG (SCBT, sc-2027) or anti-p53 antibody (rabbit polyclonal, Diagenode C15410083). We used magnetic protein G beads (Dynabeads, Invitrogen 10003D) for immunoprecipitations. Inputs correspond to 1% of the total DNA sample. We de-crosslinked the inputs and immunoprecipitates and purified DNA with MicroChIP DiaPure columns (Diagenode C03040001). We analyzed TINCR p53 RE enrichment over the input chromatin by quantitative real-time PCR (Applied Biosystems) using FastStart Universal SYBR Green (ROCHE) with the following primers: P53 RE TINCR int1 Fw 5′-CAACATGGTGAAACCCCATC-3′, and P53 RE TINCR int1 Rv 5′-CGCCTCCCAGACTCAAG-3′.

### Mouse organs histopathology

We fixed mouse organs in 10% buffered formalin. The Molecular Pathology shared resource facility at the Herbert Irving Cancer Comprehensive Center proceeded to embed fixed mouse organs in paraffin blocks, sectioning, and hematoxylin and eosin (hematoxylin-eosin) stain by following standard procedures. All slides were digitalized on a Leica SCN 400.

### Squamous stratified epithelia analyses

We quantified the thickness of the epidermis from hematoxylin-eosin-stained back and tail skin samples by taking the average of three randomly selected skin regions (six measures per region) per mouse. In the case of squamous stratified epithelia from the esophagus and forestomach, we quantified thickness by taking the average of 25 measures from one large randomly selected area per organ and mouse. We counted manually the number of cells found in the differentiated layers from the tail skin, esophagus, and forestomach, and normalized this calculation per area (mm^2^).

### Patients and tissue specimens

We collected surgical tissue specimens from HNSCC patients who underwent resection of their tumors at the Hospital Universitario Central de Asturias between 1990 and 2010, with written informed consent in accordance with approved institutional review board guidelines. The formalin-fixed, paraffin-embedded tissue samples and data from donors included in this study were provided by the Principado de Asturias BioBank (PT17/0015/0023), integrated into the Spanish National Biobanks Network, and they were processed following standard operating procedures with the appropriate approval of the Ethical and Scientific Committees.

A homogenous cohort of 306 surgically treated HNSCC patients was selected for study according to the following criteria: (a) having a single primary surgically treated tumor in the oropharynx, hypopharynx, or larynx; (b) confirmed microscopically clear surgical margins; (c) no treatments prior to surgery; (d) a minimum follow-up of 5 years. The main clinical and pathological features are summarized in Supplementary Data 11. Information on HPV status was available for all the patients. HPV detection was performed using p16 immunohistochemistry, high-risk HPV DNA detection by in situ hybridization, and genotyping by GP5+/6+ -PCR, as previously reported^40,41^.

The Department of Pathology electronic database at Hospital Universitario Central de Asturias was searched to locate all the patients who had developed nodal metastases from cSCC of the head and neck (cSCCHN) during 1998–2008. All the electronic medical records were reviewed (by Drs. García-Pedrero and Santos-Juanes), and information regarding clinical variables, date of nodal metastasis, and death were collected. A total of 50 patients with primary cSCCHN were included who had histologically confirmed lymph node metastasis. Controls (50 patients) were randomly selected among those patients with cSCCHN who did not have any metastases and had a minimum follow-up of 4 years.

All the tumors were excised by wide local excision with ≥5-mm margins. Patients with positive margins were excluded. None of the patients received any form of adjuvant therapy after their surgery. Ethics approval was obtained from the Hospital Universitario Central de Asturias ethics committee. The study was conducted and the results were reported according to the Strengthening the Reporting of Observational Studies in Epidemiology guidelines for case-control studies. Clinical patient-related data were collected retrospectively. Patient age was defined as the age at the time of resection. Pathologic tumor staging was based on the seventh American Joint Committee on Cancer classification. Outcome data were from patients with one tumor.

A commercial cutaneous SCC with adjacent normal skin tissue array (SK801c) containing 67 tumor samples and nine normal skin samples was from US Biomax Inc.

### Head and neck and cutaneous SCC tissue microarray (TMA) construction and immunohistochemistry

Three morphologically representative areas (1 mm diameter cylinders) were selected from each individual tumor paraffin block for the construction of tissue microarrays (TMAs) as described previously^40^. In addition, each TMA included three cores of the normal epithelium (tonsillar, pharyngeal, and laryngeal mucosa obtained from non-oncologic patients) as an internal negative control. The TMAs were cut into 3 μm sections and dried on Flex IHC microscope slides (Dako, Glostrup, Denmark). The sections were deparaffinized with standard xylene and hydrated through graded alcohols into water. Antigen retrieval was performed using Envision Flex Target Retrieval solution, high pH (Dako). Staining was done at room temperature on an automatic staining workstation (Dako Autostainer Plus, Dako, Glostrup, Denmark) using TINCR rabbit polyclonal antibody at 1:1000 dilution. Immunodetection was carried out with the Dako EnVision Flex + Visualization System (Dako Autostainer, Dako, Glostrup, Denmark), using diaminobenzidine as chromogen. Counterstaining with hematoxylin was the final step. A sample of normal skin was used as a positive control. Negative controls with an omission of the antiserum from the primary incubation were also included.

To quantify TINCR expression, a semiquantitative scoring system based on staining intensity was applied, divided into three categories: negative (absence of staining, score 0), weak to moderate (some cytoplasmic staining in tumor areas, score 1), and strong protein expression (intense and homogeneous cytoplasmic staining in tumor areas, score 2), with an inter-observer concordance higher than 95%. For statistical purposes, TINCR staining was dichotomized as positive expression (score 1–2) versus negative or loss of TINCR expression (score 0).

### Fluorescence in situ hybridization (FISH)

We used two FISH probes to study the TINCR locus. RP11-565J3 specific bacterial artificial chromosome (BACs) that map at the TINCR locus (19p13.3) and RP11-937M15 that map to a control region (19q13.11) were purchased from the Human BAC Clone Library, Children’s Hospital Oakland Research Institute (CHORI) and labeled by Nick translation assay with FITC and Texas-Red, respectively, to generate locus-specific FISH probes. We used the RP11-937M15 BAC clone to generate a control probe to enumerate chromosome 19. FISH analyses were performed according to the manufacturer’s instructions, as previously described in ref. ^42^ on 5 mm TMA sections mounted on positively charged slides (SuperFrost, Thermo Scientific). Briefly, the slides were first deparaffined in xylene and rehydrated gradually in a series of ethanol (70, 80, 95%). We used the Histology FISH Accessory Kit (DAKO) following the manufacturer’s instructions. Briefly, we pre-treated in 2-[*N*-morpholino]ethanesulphonic acid (MES), followed by protein digestion performed in a pepsin solution. After dehydration, we denatured the samples in the presence of the specific probe at 66 °C for 10 min and left them overnight for hybridization at 45 °C in a DAKO hybridizer machine. Finally, we washed the slides with 20×SSC (saline-sodium citrate) buffer with detergent Tween-20 at 63 °C and mounted them in a fluorescence mounting medium (DAPI). We manually enumerated FISH signals within nuclei all over the tissue. FISH images were also captured using a CCD camera (Photometrics SenSys camera) connected to a PC running the Zytovision image analysis system (Applied Imaging Ltd., UK) with a focus motor and Z stack software.

### Mutation analysis of tumor samples

We performed dideoxynucleotide DNA sequencing of PCR products encompassing the *TINCR* coding exons in DNA extracted from formalin-fixed paraffin-embedded HNSCC samples using standard procedures at Genewiz (South Plainfield, New Jersey). Mutations were identified using Alignments (Benchling, San Francisco, California) and manual curation of sequencing chromatograms. To detect mutations in *Trp53* from UVB-induced mouse lesions, we performed dideoxynucleotide DNA sequencing of PCR products encompassing the *Trp53* coding exons in DNA-extracted formalin-fixed paraffin-embedded mouse papilloma and squamous cell carcinoma samples using standard procedures at Genewiz (South Plainfield, New Jersey).

### TINCR recombinant protein production and purification

We amplified the human TINCR coding sequence by PCR from pcDNA 3.1-TESO and cloned it in-frame following an N-terminal hexahistidine (His_6_) tag in *BseR*I linearized pET28a-LIC expression vector (Addgene 26094). We expressed TINCR recombinant protein from Rosetta 2 (DE3) *Escherichia coli* cells by induction with 0.5 mM isopropyl-b-d-thiogalactopyranoside 3 h at 37 °C. We resuspended cells in lysis buffer (50 mM HEPES pH 7.5, 500 mM sodium chloride, 10% glycerol, 0.5 mM TECP, 20 mM imidazole) supplemented with complete EDTA-free protease inhibitor (Roche) and lysed cells by sonication. We purified TINCR recombinant protein using an AKTA fast protein liquid chromatography system (GE Healthcare). We first performed affinity chromatography using a 1 mL Nickel-charged HisTrap column (GE Healthcare) in a step-wise method with elution buffer (lysis buffer with 500 mM imidazole) by first setting the buffer ratio to 25% elution buffer for eight column volumes, and then switching to a linear gradient to 100% elution buffer over 10 column volumes. We pooled TINCR-containing fractions and purified further by size exclusion chromatography using a Hi Load 16/60 Superdex 200 gel filtration column (GE Healthcare) equilibrated in 50 mM HEPES pH 7.5, 100 mM NaCl, 10% glycerol, and 0.5 mM TCEP. We assessed protein expression and purity by SDS-PAGE and Coomassie staining and concentrated protein samples to ~5–6 mg/ml.

### Crystallization and structure determination

The human TINCR protein at a concentration of 5.5 mg/ml in a protein buffer (20 mM HEPES (pH 7.5), 100 mM sodium chloride, 5% (v/v) glycerol, and 1 mM TCEP) was initially subjected to extensive robotic screening at the High-Throughput Crystallization Screening Center of the Hauptman-Woodward Medical Research Institute (HWI) (https://hwi.buffalo.edu/high-throughput-crystallization-center/)^43^. For the first week, the crystallization plate was placed in a 4 °C incubator, during which six crystal hits were detected by manually checking the robotically-taken images. All six crystallization conditions were initially used to set up an in-house crystallization plate using the micro-batch, under oil, method in a cold room (4 °C). Very small rod-like crystals of the TINCR protein appeared after a week in all conditions. Further crystal optimization was performed using the seeding method. The largest clusters of rod-like crystals appeared in a condition comprising 0.1 M magnesium sulfate heptahydrate, 0.1 M sodium acetate, pH 5, and 20% (w/v) PEG 1000, after a few days. A single crystal with dimensions approximately 200 µm long and 10 µm wide was transferred to the aforementioned crystallization condition, which was already supplemented with 20% (v/v) glycerol as a cryo-protectant, and flash-frozen in liquid nitrogen for the subsequent data collection at the NE-CAT 24-ID-E beamline of Advanced Photon Source in Lemont, IL. The TINCR crystal diffracted the X-ray beam to a resolution of 2.12 Å. The images were processed and scaled in space group *P*4_3_2_1_2 using XDS^44^. The structure of TINCR was determined by molecular replacement method using both MOLREP^45^ and PHASER^46^ programs. The first model, generated by the protein fold recognition server Phyre2^47^ from the crystal structure of the ubiquitin-like domain of the BAG protein (PDB id: 4HWI^48^) from Arabidopsis thaliana, with 21% sequence identity with that of TINCR was successfully used as a search model for structural determination of the ubiquitin-like domain of the TINCR protein. The N-terminal 15 amino acids of the TINCR protein, which forms an α-helical cap, was subsequently modeled by programs XtalView^49^ and Coot^50^ and refined using both CNS^51^ and Phenix^52^ programs. The refined structure reveals that there are two TINCR protomers, forming one dimer, in the asymmetric unit of the crystal. The a-helical cap is visible in one protomer and it is highly mobile as inferred by its high B-factor values. The crystal structure of the TINCR protein has been deposited in Protein Data Bank with accession code 7MRJ. Crystallographic statistics are shown in Supplementary Data 6, 7.

### Modeling

A structural model was generated for each of five TINCR mutants (R5W, G7K, T30I, A42T, and V18M-V49M) using Coot^50^, followed by refinement by Phenix^52^ against the TINCR wild-type dataset. In each case, the resulting model was compared with those generated by modeling servers Phyre2^47^ and iTASSER^53^. Electrostatic surface potential was calculated using APBS^54^ and visualized in PyMOL (https://pymol.org/2/).

### Colony formation assay

We plated 500 cells (CAL-27 and FaDu) at a single-cell suspension into 12-well plates containing a complete medium. We cultured cells for 2 weeks with the medium being replaced every 3–5 days. Then, we fixed cells with 4% formaldehyde, stained them with 0.1% crystal violet, and scanned for quantification of colonies with FIJI ImageJ. We used 10% acetic acid to dissolve incorporated crystal violet and we measure absorbance at an optical density of 590 nm in a plate reader (Biorad).

### Tumor xenografts

Female 6-week-old Nude (NU/NU [088] Charles River) mice were used for orthotopic transplantations and xenograft studies. Human HNSCC cell lines (CAL-27 and FaDu) were suspended in 50% Matrigel (BD, 356237) diluted with DMEM (SIGMA, #H0135) media at a concentration of 50,000 cells per inoculation, and injected intradermally into Nude (Charles River, NU/NU [088]) recipient mice. Tumors were detected by palpation, measured using a digital caliper, and tumor volume was calculated (V_Tumor_ = $\frac{\pi }{6}$xlxw^2^, where l = length in mm and w = width in mm).

### Statistics

For patient tumor sample analysis, *χ*^2^ and Fisher’s exact tests were used for comparison between categorical variables. For time-to-event analysis, Kaplan–Meier curves were plotted. Differences between survival times were analyzed by the log-rank method. All tests were two-sided. *p* values of ≤0.05 were considered statistically significant. We conducted statistical analyses using Prism software v8.0 (GraphPad Software).

#### Study approval

All experimental procedures were conducted in accordance with the Declaration of Helsinki and approved by the Institutional Ethics Committee of the Hospital Universitario Central de Asturias and by the Regional CEIm from Principado de Asturias (date of approval May 14, 2019; approval number: 141/19) for the project PI19/00560. Animal studies were conducted under the supervision of the Columbia University Irving Medical Center IACUC.

### Reporting summary

Further information on research design is available in the Nature Portfolio Reporting Summary linked to this article.

## Supplementary information

Supplementary Information

Description of Additional Supplementary Files

Supplementary Data 1

Supplementary Data 2

Supplementary Data 3

Supplementary Data 4

Supplementary Data 5

Supplementary Data 6

Supplementary Data 7

Supplementary Data 8

Supplementary Data 9

Supplementary Data 10

Supplementary Data 11

Reporting Summary

## Data Availability

RNA-sequencing data analyzed here from Cancer Genome Atlas (TCGA) database is available in the database of Genotypes and Phenotypes (dbGaP) under the accession number phs000178. The protein structure for TINCR have been deposited in the Protein Data Bank (PDB) with the accession code 7MRJ. Source data are provided with this paper.

## Source data

Source Data
