# Supplementary material for: The TINCR ubiquitin-like microprotein is a tumor suppressor in squamous cell carcinoma
Source: Nat Commun. 2023 Mar 10;14:1328. doi: 10.1038/s41467-023-36713-8 (PMC10006087; doi:10.1038/s41467-023-36713-8)
Supplement: Supplementary file 1 — Supplementary Information [file 41467_2023_36713_MOESM1_ESM.pdf]

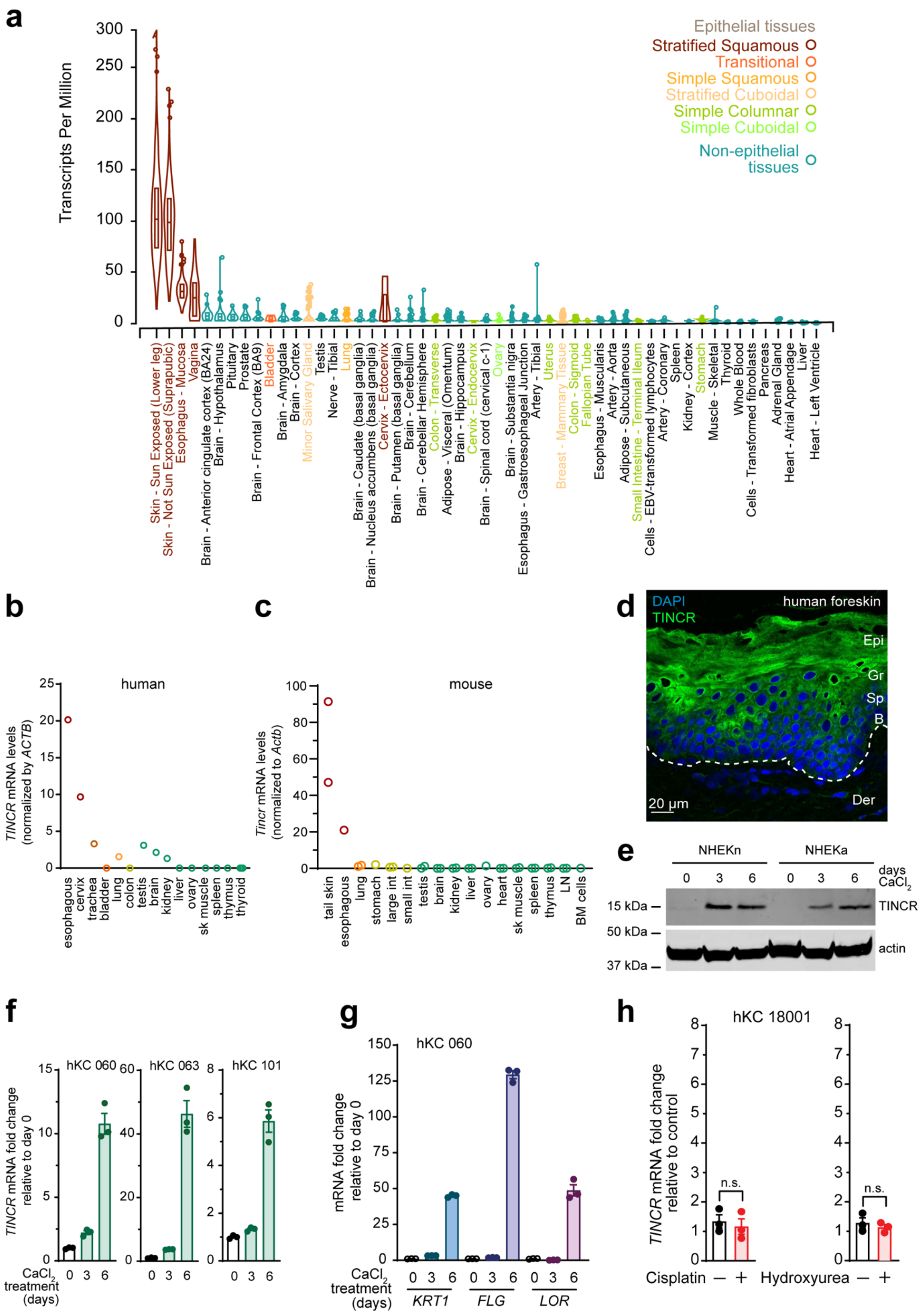

a
Transcripts Per Million
Epithelial tissues
Stratified Squamous
Transitional
Simple Squamous
Stratified Cuboidal
Simple Columnar
Simple Cuboidal
Non-epithelial tissues
b
human
TINCR mRNA levels (normalized by ACTB)
c
mouse
Tincr mRNA levels (normalized to Actb)
d
DAPI
TINCR
human foreskin
Epi
Gr
Sp
B
Der
20 μm
e
NHEKn
NHEKa
days CaCl2
15 kDa
50 kDa
37 kDa
TINCR
actin
f
hKC 060
hKC 063
hKC 101
TINCR mRNA fold change relative to day 0
CaCl2 treatment (days)
g
hKC 060
mRNA fold change relative to day 0
KRT1
FLG
LOR
h
hKC 18001
TINCR mRNA fold change relative to control
n.s.
Cisplatin
Hydroxyurea

Supplementary Figure 1. Stratified epithelia-specific expression of *TINCR* RNA and TINCR protein. a, Violin plot representation of *TINCR* transcript expression assessed by RNAseq in GTEx human tissues. **b,** Quantitative RT-PCR expression of relative *TINCR* mRNA expression in human tissues. Mean values of technical triplicates are shown. **c,** Quantitative RT-PCR expression of relative *Tincr* mRNA expression in mouse tissues. Mean values of technical replicates in tissues from two independent mice are shown. **d,** Immunofluorescence detection of TINCR protein expression in human foreskin. Cell nuclei are stained with DAPI, TINCR protein is shown in green. Der:dermis, B: basal layer, Sp: spinocellular layer, Gr: glanulose layer, Epi: epidermis. Dashed line indicates the dermal-epidermal boundary. **e,** Western blot analysis of endogenous TINCR microprotein in neonatal (NHEKn) and adult (NHEKa) normal human keratinocytes upon *in vitro* differentiation following calcium chloride treatment. **f,** Quantitative RT-PCR analysis of *TINCR* expression in primary human keratinocytes isolated from 3 independent foreskin samples (060, 061, and 101) upon *in vitro* differentiation following calcium chloride treatment. Graphs show median gene expression values normalized to *ACTB* and represented as fold change relative to day 0. Error bars correspond to standard error of the mean across three technical replicas. **g,** Expression of differentiation marker genes *KRT1*, *FLG* and *LOR* analyzed by quantitative RT-PCR in primary human keratinocytes (060) following calcium chloride induced differentiation *in vitro* at indicated time points. Bar graphs show median gene expression values normalized to *ACTB* and represented as fold change relative to day 0. Error bars correspond to standard error of the mean across three technical replicas. **h,** Quantitative RT-PCR analysis of *TINCR* expression in primary human keratinocytes isolated from foreskin samples (18001) in basal conditions and 24 hours following Cisplatin (20uM) or Hydroxyurea (2mM) treatment. Graphs show average values normalized to *ACTB* relative to untreated controls. Error bars indicate standard error of the mean in technical replicates. P values correspond to two-tailed unpaired Student's t-test. Source data are provided as a Source Data file.

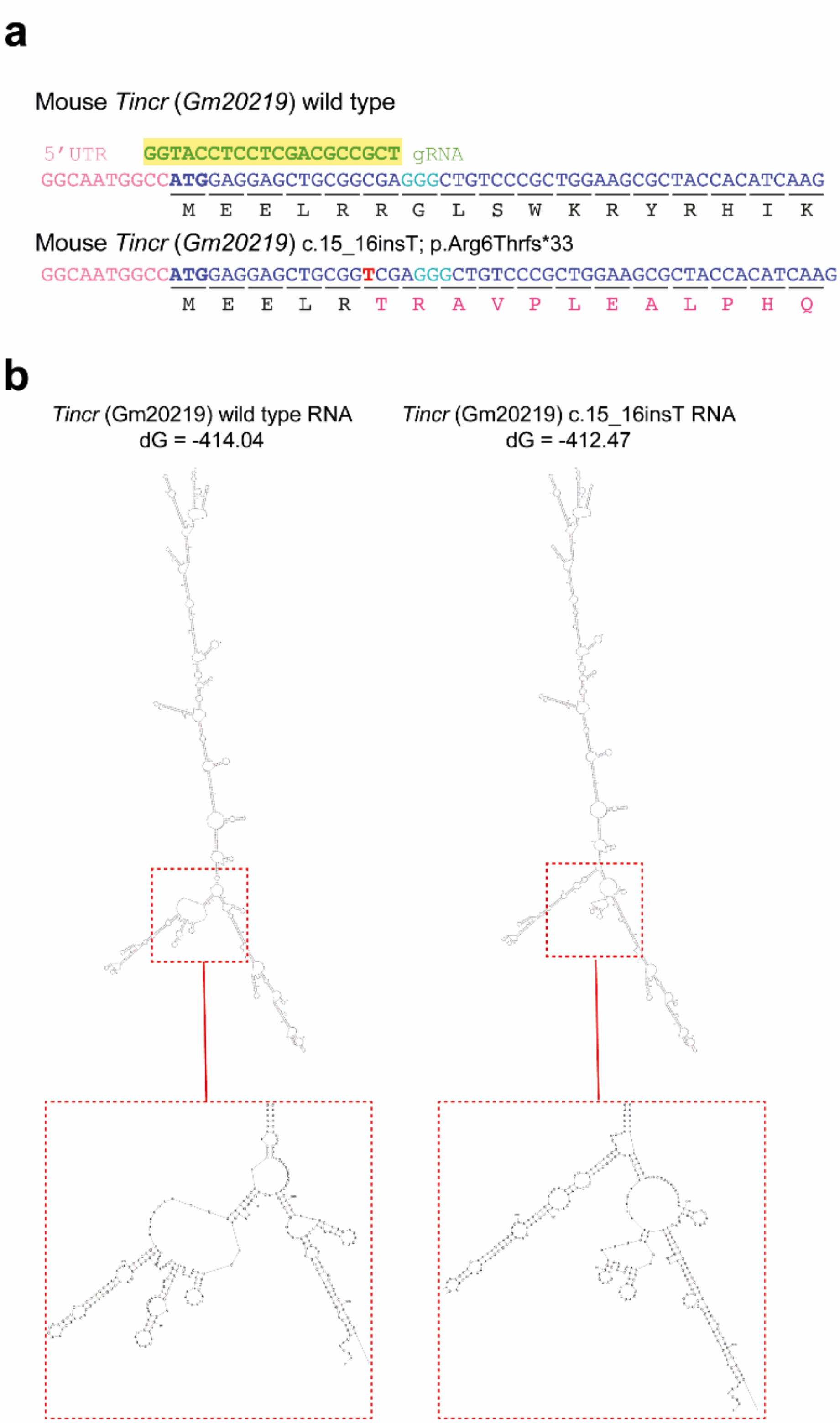

Supplementary Figure 2. Generation of *Tincr* mutant mice. a, Partial nucleotide and predicted amino acid sequences of the wild type mouse *Tincr* and *Tincr* p.Arg6Thrfs*33 mutant. PAM sequence (cyan GGG) and gRNA used for mutagenesis are indicated. **b**, Predicted secondary structure of wild type and *Tincr* p.Arg6Thrfs*33 mutant transcribed RNA.

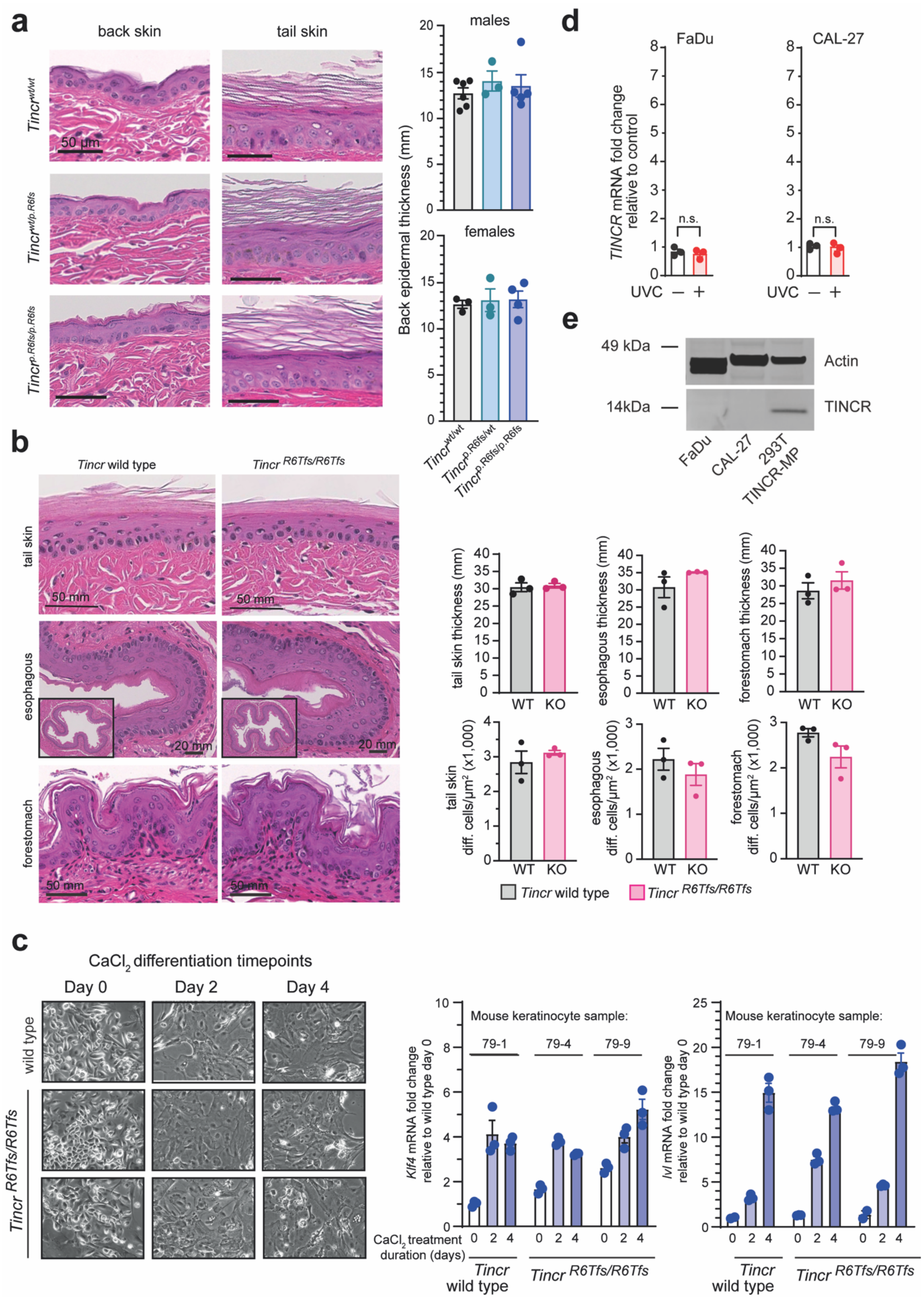
a
back skin
tail skin
Tincr wt/wt
Tincr wt/p.R6fs
Tincr p.R6fs/p.R6fs
50 µm
males
females
Back epidermal thickness (mm)
Tincr wt/wt
Tincr p.R6fs/wt
Tincr p.R6fs/p.R6fs
d
FaDu
CAL-27
TINCR mRNA fold change relative to control
n.s.
UVC – +
e
49 kDa
14kDa
Actin
TINCR
FaDu
CAL-27
293T TINCR-MP
b
Tincr wild type
Tincr R6Tfs/R6Tfs
tail skin
esophagous
forestomach
50 mm
20 mm
tail skin thickness (mm)
esophagous thickness (mm)
forestomach thickness (mm)
tail skin diff. cells/µm² (x1,000)
esophagous diff. cells/µm² (x1,000)
forestomach diff. cells/µm² (x1,000)
WT KO
Tincr wild type
Tincr R6Tfs/R6Tfs
c
CaCl2 differentiation timepoints
Day 0
Day 2
Day 4
wild type
Tincr R6Tfs/R6Tfs
Mouse keratinocyte sample:
79-1
79-4
79-9
Klf4 mRNA fold change relative to wild type day 0
Ivl mRNA fold change relative to wild type day 0
CaCl2 treatment duration (days)
0 2 4
Tincr wild type
Tincr R6Tfs/R6Tfs

Supplementary Figure 3. Analysis of *Tincr* mutant mice. a. Representative images of hematoxylin-eosin stained back and tail skin samples from *Tincr* wild type, heterozygous and homozygous knockout mice. Quantification of epidermal thickness in male and female *Tincr* wild type, heterozygous and homozygous knockout mice. Each dot represents one mouse, graphs correspond to average levels and error bars indicate the standard error of the mean. **b**, Representative hematoxylin-eosin stained micrographs of tail skin, esophagus and forestomach tissues from *Tincr* wild type and *Tincr* p.Arg6Thrfs*33 homozygous mutant and quantification of epidermal thickness and cellularity in differentiated epidermal layers in tail skin, esophagus and forestomach tissue samples from *Tincr* wild type and *Tincr* p.Arg6Thrfs*33 homozygous mutant mice (n=3 per genotype). **c**, Representative images of *Tincr* wild type and *Tincr* p.Arg6Thrfs*33 homozygous mutant mouse keratinocytes following calcium chloride *in vitro* differentiation. Quantitative RT-PCR analysis of the *Klf4* early keratinocyte differentiation marker in one wild type and two independent *Tincr* knockout primary mouse keratinocyte samples upon *in vitro* calcium chloride-induced differentiation. Graphs show average *Klf4* RNA levels normalized to *Actb* and represented as fold change relative to wild type mouse keratinocytes at day 0 of calcium chloride treatment. **d,** Quantitative RT-PCR analysis of *TINCR* expression in FaDu and CAL-27 cell lines in basal conditions and 4 hours following UVC radiation (20mJ/cm^2). Graphs show average values normalized to *ACTB* relative to untreated controls. Error bars indicate standard error of the mean in technical replicates. P values correspond to two-tailed unpaired Student's t-test. **e,** Western blot analysis of endogenous TINCR microprotein in FaDu, CAL-27 and 293T cells expressing TINCR-microprotein (MP). Source data are provided as a Source Data file.

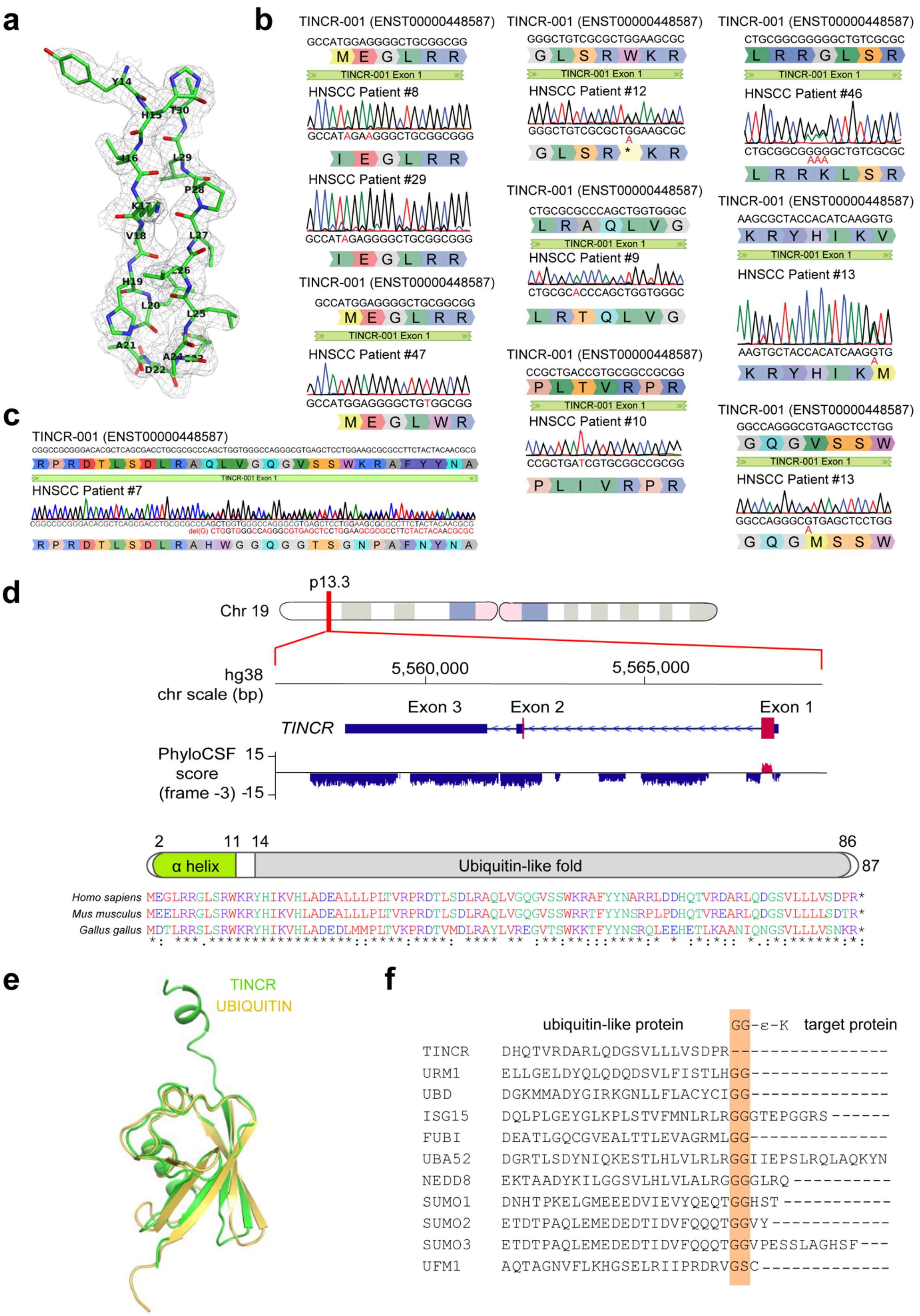
a
Y14
H15
T30
I16
L29
P28
K17
V18
L27
L26
H19
L20
L25
A21
A24
D22
b
TINCR-001 (ENST00000448587)
GCCATGGAGGGGCTGCGGCGG
M E G L R R
TINCR-001 Exon 1
HNSCC Patient #8
GCCATAGAAGGGCTGCGGCGGG
I E G L R R
HNSCC Patient #29
GCCATAGAGGGGCTGCGGCGGG
I E G L R R
TINCR-001 (ENST00000448587)
GCCATGGAGGGGCTGCGGCGG
M E G L R R
TINCR-001 Exon 1
HNSCC Patient #47
GCCATGGAGGGGCTGTGGCGG
M E G L W R
TINCR-001 (ENST00000448587)
GGGCTGTCGCGCTGGAAGCGC
G L S R W K R
TINCR-001 Exon 1
HNSCC Patient #12
GGGCTGTCGCGCTGGAAGCGC
A
G L S R * K R
TINCR-001 (ENST00000448587)
CTGCGCGCCCAGCTGGTGGGC
L R A Q L V G
TINCR-001 Exon 1
HNSCC Patient #9
CTGCGCACCCAGCTGGTGGGC
L R T Q L V G
TINCR-001 (ENST00000448587)
CCGCTGACCGTGCGGCCGCGG
P L T V R P R
TINCR-001 Exon 1
HNSCC Patient #10
CCGCTGATCGTGCGGCCGCGG
P L I V R P R
TINCR-001 (ENST00000448587)
CTGCGGCGGGGGCTGTCGCGC
L R R G L S R
TINCR-001 Exon 1
HNSCC Patient #46
CTGCGGCGGGGGCTGTCGCGC
AAA
L R R K L S R
TINCR-001 (ENST00000448587)
AAGCGCTACCACATCAAGGTG
K R Y H I K V
TINCR-001 Exon 1
HNSCC Patient #13
AAGTGCTACCACATCAAGGTG
A
K R Y H I K M
TINCR-001 (ENST00000448587)
GGCCAGGGCGTGAGCTCCTGG
G Q G V S S W
TINCR-001 Exon 1
HNSCC Patient #13
GGCCAGGGCGTGAGCTCCTGG
A
G Q G M S S W
c
TINCR-001 (ENST00000448587)
CGGCCGCGGGACACGCTCAGCGACCTGCGCGCCCAGCTGGTGGGCCAGGGCGTGAGCTCCTGGAAGCGCGCCTTCTACTACAACGCG
R P R D T L S D L R A Q L V G Q G V S S W K R A F Y Y N A
TINCR-001 Exon 1
HNSCC Patient #7
del(G)
R P R D T L S D L R A H W G G Q G G T S G N P A F N Y N A
d
p13.3
Chr 19
hg38
chr scale (bp)
5,560,000
5,565,000
Exon 3
Exon 2
Exon 1
TINCR
PhyloCSF score (frame -3)
15
-15
2
11
14
86
87
α helix
Ubiquitin-like fold
Homo sapiens MEGLRRGLSRWKRYHIKVHLADEALLLPLTVRPRDTLSDLRAQLVGQGVSSWKRAFYYNARRLDDHQTVRDARLQDGSVLLLVSDPR*
Mus musculus MEELRRGLSRWKRYHIKVHLADEALLLPLTVRPRDTLSDLRAQLVGQGVSSWRRTFYYNSRPLPDHQTVREARLQDGSVLLLLSDTR*
Gallus gallus MDTLRRSLSRWKRYHIKVHLADEDLMMPLTVKPRDTVMDLRAYLVREGVTSWKKTFYYNSRQLEEHETLKAANIQNGSVLLLVSNKR*
e
TINCR
UBIQUITIN
f
ubiquitin-like protein
GG-ε-K
target protein
TINCR DHQTVRDARLQDGSVLLLVSDPR
URM1 ELLGELDYQLQDQDSVLFISTLHGG
UBD DGKMMADYGIRKGNLLFLACYCIGG
ISG15 DQLPLGEYGLKPLSTVFMNLRLRGGGTEPGGRS
FUBI DEATLGQCGVEALTTLEVAGRMLGG
UBA52 DGRTLSDYNIQKESTLHLVLRLRGGIIEPSLRQLAQKYN
NEDD8 EKTAADYKILGGSVLHLVLALRGGGGLRQ
SUMO1 DNHTPKELGMEEEDVIEVYQEQTGGHST
SUMO2 ETDTPAQLEMEDEDTIDVFQQQTGGVY
SUMO3 ETDTPAQLEMEDEDTIDVFQQQTGGVPESSLAGHSF
UFM1 AQTAGNVFLKHGSELRIIPRDRVGSC

Supplementary Figure 4. Structural characterization of the TINCR ubiquitin domain-containing microprotein. a, Representative electron density map for TINCR. A refined model of residues Y14-T30 depicted with stick models (green), which is fitted into 2Fo-Fc electron density map of the region contoured at 1 σ (grey mesh). **b,** Chromatograms depicting point mutations in HNSCC patient tumor samples within the first exon of *TINCR*. Single nucleotide variants are highlighted in red. **c**, Chromatogram depicting a single nucleotide frameshift insertion detected in a HNSCC patient tumor sample within the first exon of *TINCR*. **d,** Schematic representation of the human *TINCR* locus, PhyloCSF conservation analysis across vertebrate species indicating a positive score for multispecies amino acid sequence conservation (red) along a sORF encoded in exons 1 and 2, and schematic domain distribution and amino acid sequence along the predicted mouse and chicken orthologs for this sORF. **e,** Structural overlay of TINCR and Ubiquitin structures. **f,** Amino acid sequence alignment of the C-terminal region of TINCR and UBL proteins. The C-terminal di-Gly motif present in most UBL proteins is highlighted in orange. ISG15, UBA52, NEDD8 and SUMO proteins undergo proteolytic cleavage that expose the di-Gly motif.

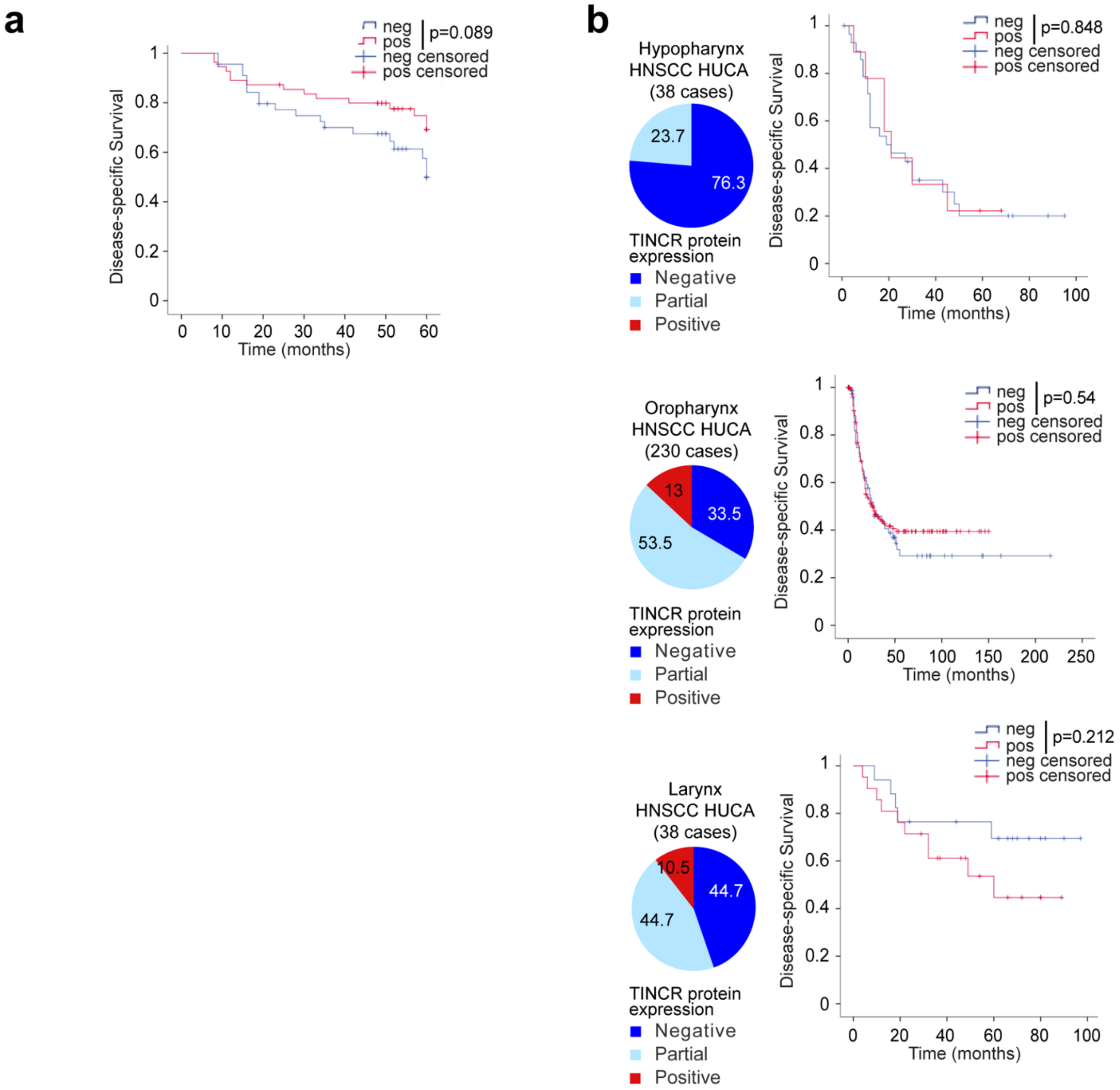

Supplementary Figure 5. Loss of TINCR protein expression in prognosis of human cancer. **a,** Kaplan-Meyer curve showing disease-specific survival of cSCC patients according to TINCR expression: negative (absence and partial expression of TINCR in differentiated areas of the tumors) or positive (diffuse cytoplasmic TINCR stain). **b,** Pie chart graphs show the percentage of cases with negative (blue), partial (light blue) or positive (red) stain of TINCR according to their anatomical location (hypopharynx, oropharynx, and larynx) in HNSCC patients. Disease-specific survival curve of HNSCC according to anatomical location is also shown. Differences between survival times were analyzed by the log-rank method (P values shown) in (**a**) and (**b**).
