# Supplementary material for: The TINCR ubiquitin-like microprotein is a tumor suppressor in squamous cell carcinoma
Source: Nat Commun. 2023 Mar 10;14:1328. doi: 10.1038/s41467-023-36713-8 (PMC10006087; doi:10.1038/s41467-023-36713-8)
Supplement: Supplementary file 2 — Description of Additional Supplementary Files [file 41467_2023_36713_MOESM2_ESM.pdf]

Description of Additional Supplementary Files

File Name: Supplementary Data 1
Description: Sex differences of UVB-induced skin carcinogenesis in mice

File Name: Supplementary Data 2
Description: TRP53 mutations in UVB-induced skin carcinogenesis of mice

File Name: Supplementary Data 3
Description: TCGA differential gene expression analysis of high and low TINCR expressing cases

File Name: Supplementary Data 4
Description: GSEA pathways upregulated in HNSCC patients with high TINCR expression

File Name: Supplementary Data 5
Description: GSEA pathways upregulated in HNSCC patients with low TINCR expression

File Name: Supplementary Data 6
Description: DALI server TINCR structural homologs (PDB all)

File Name: Supplementary Data 7
Description: Crystallographic data collection and refinement statistics

File Name: Supplementary Data 8
Description: TINCR expression in head and neck squamous cell carcinomas

File Name: Supplementary Data 9
Description: TINCR expression in cutaneous squamous cell carcinoma and other skin cancers

File Name: Supplementary Data 10
Description: Association of TINCR protein expression with other proteins by immunohistochemistry and clinical parameters in HUCA cutaneous SCC cohort

File Name: Supplementary Data 11
Description: Association of TINCR protein expression with clinicopathological findings, relapse and disease outcome
