# Supplementary material for: The TINCR ubiquitin-like microprotein is a tumor suppressor in squamous cell carcinoma
Source: Nat Commun. 2023 Mar 10;14:1328. doi: 10.1038/s41467-023-36713-8 (PMC10006087; doi:10.1038/s41467-023-36713-8)
Supplement: Supplementary file 14 — Reporting Summary [file 41467_2023_36713_MOESM14_ESM.pdf]

nature portfolio

Corresponding author(s): Adolfo Ferrando

Last updated by author(s): Jan 6, 2023

Reporting Summary

Nature Portfolio wishes to improve the reproducibility of the work that we publish. This form provides structure for consistency and transparency in reporting. For further information on Nature Portfolio policies, see our Editorial Policies and the Editorial Policy Checklist.

Statistics

For all statistical analyses, confirm that the following items are present in the figure legend, table legend, main text, or Methods section.

n/a	Confirmed	
☐	☒	The exact sample size (*n*) for each experimental group/condition, given as a discrete number and unit of measurement
☐	☒	A statement on whether measurements were taken from distinct samples or whether the same sample was measured repeatedly
☐	☒	The statistical test(s) used AND whether they are one- or two-sided<br>*Only common tests should be described solely by name; describe more complex techniques in the Methods section.*
☒	☐	A description of all covariates tested
☐	☒	A description of any assumptions or corrections, such as tests of normality and adjustment for multiple comparisons
☐	☒	A full description of the statistical parameters including central tendency (e.g. means) or other basic estimates (e.g. regression coefficient) AND variation (e.g. standard deviation) or associated estimates of uncertainty (e.g. confidence intervals)
☐	☒	For null hypothesis testing, the test statistic (e.g. *F*, *t*, *r*) with confidence intervals, effect sizes, degrees of freedom and *P* value noted<br>*Give P values as exact values whenever suitable.*
☒	☐	For Bayesian analysis, information on the choice of priors and Markov chain Monte Carlo settings
☒	☐	For hierarchical and complex designs, identification of the appropriate level for tests and full reporting of outcomes
☒	☐	Estimates of effect sizes (e.g. Cohen's *d*, Pearson's *r*), indicating how they were calculated

Our web collection on statistics for biologists contains articles on many of the points above.

Software and code

Policy information about availability of computer code

Data collection	RNAseq data was obtained from the database of Genotypes and Phenotypes (dbGaP) under accession number phs000178
Data analysis	Reads were aligned to the human (GRCh38) genome and gene-count matrices were evaluated for differential gene expression in cBioPortal We conducted statistical analyses using Prism software v8.0 (GraphPad Software). Structural model was generated for each of five TINCR mutants (R5W, G7K, T30I, A42T, and V18M-V49M) using Coot, followed by refinement by Phenix against the TINCR wild-type dataset. In each case, the resulting model was compared with those generated by modeling servers Phyre247 and iTASSER53. Electrostatic surface potential was calculated using APBS54 and visualized in PyMOL (https://pymol.org/2/).

For manuscripts utilizing custom algorithms or software that are central to the research but not yet described in published literature, software must be made available to editors and reviewers. We strongly encourage code deposition in a community repository (e.g. GitHub). See the Nature Portfolio guidelines for submitting code & software for further information.

Data

Policy information about availability of data

All manuscripts must include a data availability statement. This statement should provide the following information, where applicable:

- Accession codes, unique identifiers, or web links for publicly available datasets
- A description of any restrictions on data availability
- For clinical datasets or third party data, please ensure that the statement adheres to our policy

RNA-sequencing data analyzed here from Cancer Genome Atlas (TCGA) database is available in the database of Genotypes and Phenotypes (dbGaP) under accession number phs000178. The protein structure for TINCR can be found under PDB code 7MRJ. Source data are provided with this paper.

Human research participants

Policy information about studies involving human research participants and Sex and Gender in Research.

Reporting on sex and gender	NA, all samples analyzed were devoid of sex and gender identifyers
Population characteristics	A homogenous cohort of 306 surgically treated HNSCC patients was selected for study according to the following criteria: a) having a single primary surgically treated tumor in oropharynx, hypopharynx or larynx; b) confirmed microscopically clear surgical margins; c) no treatments prior to surgery; d) a minimum follow-up of five years.<br><br>The formalin-fixed, paraffin-embedded tissue samples and data from donors included in this study were provided by the Principado de Asturias BioBank (PT17/0015/0023), integrated in the Spanish National Biobanks Network, and they were processed following standard operating procedures with the appropriate approval of the Ethical and Scientific Committees. Samples were obtained with written informed consent. Analysis was conducted under the supervision of the Columbia University Medical Center Institutional Review Board. All samples were deidentified and provided without associated demographic data (age, sex, ethnicity).
Recruitment	Patients were included in the study based on tumor sample availability
Ethics oversight	The formalin-fixed, paraffin-embedded tissue samples and data from donors included in this study were provided by the Principado de Asturias BioBank (PT17/0015/0023), integrated in the Spanish National Biobanks Network, and they were processed following standard operating procedures with approval of the Ethical and Scientific Committee. The Columbia University Medical Center Institutional Review Board approved human sample collection and analysis. The Columbia University Medical Center Institutional Review Board approved human sample collection and analysis. Approved IRB study protocol #AAAB3250

Note that full information on the approval of the study protocol must also be provided in the manuscript.

Field-specific reporting

Please select the one below that is the best fit for your research. If you are not sure, read the appropriate sections before making your selection.

☒ Life sciences ☐ Behavioural & social sciences ☐ Ecological, evolutionary & environmental sciences

For a reference copy of the document with all sections, see nature.com/documents/nr-reporting-summary-flat.pdf

Life sciences study design

All studies must disclose on these points even when the disclosure is negative.

Sample size	Sample size was chosen based on previous experience with similar experiments ()e.g. Perez garcia et al Blood . 2013 Oct 3;122(14):2425-32. doi: 10.1182/blood-2013-05-500850. , experimental feasibility, the maximal availability of samples, and N necessary to obtain difference between the groups at sufficiently high significance (p<0.05) by one-side Fisher's exact test (sample size of >15 animals per group yields >80% power to observe differences in tumor incidence >50%).
Data exclusions	No data was excluded.
Replication	All experiments were performed in at least three independent replicates as indicated in the figure legends and methods.
Randomization	Mice were allocated to different experimental groups based on their specific genotype as this was the variable under study.
Blinding	UV treatment and evaluation of tumor development was conducted blinded with respect of genotype.

Reporting for specific materials, systems and methods

We require information from authors about some types of materials, experimental systems and methods used in many studies. Here, indicate whether each material, system or method listed is relevant to your study. If you are not sure if a list item applies to your research, read the appropriate section before selecting a response.

Materials & experimental systems

n/a	Involved in the study
☐	☒ Antibodies
☐	☒ Eukaryotic cell lines
☒	☐ Palaeontology and archaeology
☐	☒ Animals and other organisms
☒	☐ Clinical data
☒	☐ Dual use research of concern

Methods

n/a	Involved in the study
☒	☐ ChIP-seq
☒	☐ Flow cytometry
☒	☐ MRI-based neuroimaging

Antibodies

Antibodies used	Immunoblotting:<br>TINCR polyclonal rabbit antibody (1:1000 dilution)<br>rabbit monoclonal antibody anti-HA tag (CST #3724, clone C29F4, 1:1000 dilution),<br>mouse monoclonal antibody anti-tubulin (SIGMA #T9026, clone DM1A, 1:5000 dilution)<br>rabbit monoclonal antibody FLAG DYKDDDDK Tag (Cell Signaling, clone D6W5B, 1:1000 dilution)<br>mouse monoclonal antibody anti-b-Actin (SIGMA #5441, clone AC-15, 1:2000 dilution)<br>Immunofluorescence:<br>TINCR polyclonal rabbit antibody (1:2000 dilution)
Validation	All antibodies were validated by the manufacturers via Wester blot analysis and immunofluorescence as documented in the manufacturers' websites.<br>TINCR polyclonal antibodies were validated by western blot analysis of cells transfected with constructs expressing tagged full length TINCR open reading frame.

Eukaryotic cell lines

Policy information about cell lines and Sex and Gender in Research

Cell line source(s)	CAL-27 (ATCC)<br>FaDu (ATCC)<br>293T-HEK (ATCC)<br>Primary human keratinocytes isolated from neonatal foreskin samples and were provided by Columbia University's Skin Stem Cell Imaging and Manipulation Core.
Authentication	Cell lines purchased from certified cell line banks or commercial vendors were not further authenticated.
Mycoplasma contamination	All lines were tested and found to be negative for mycoplasma (Mycoalert - Lonza).
Commonly misidentified lines (See ICLAC register)	We did not use any commonly misidentified lines.

Animals and other research organisms

Policy information about studies involving animals; ARRIVE guidelines recommended for reporting animal research, and Sex and Gender in Research

Laboratory animals	The following genetically modified strains were used:<br>TINCR knockout (B6; Tincr p.R6fs/p.R6fs)mice were generated at the Herbert Irving Comprehensive Cancer Center Transgenic Shared resource.<br>XPC (B6; 129-Xpctm1Ecf/J) were purchased from the Jackson Laboratory (Strain #010563).<br>Animals were housed in a controlled environment at 20-24C temperature, 45-56% humidity and 12 hour light-dark cycles.
Wild animals	The study did not involve wild animals.
Reporting on sex	When possible we analyzed similar numbers of male vs female animals. For epidermal morphology before and after UV exposure we analyzed 3 TINCR WT males, 3 TINCR het males 3 TINCR KO males, 3 TINCR WT females, 3 TINCR het females and 3 TINCR KO females. For the UVB carcinogenesis experiment, we analyzed 6 XPC het TINCR WT males, 11 XPC het TINCR WT females, 10 XPC het TINCR KO males and 13 XPC het TINCR KO females.

Field-collected samples	This study did not involve samples collected from the field.
Ethics oversight	All animals were maintained at the Irving Cancer Research Center at Columbia University Medical Campus in specific pathogen-free facilities. Mice were fed a standard chow diet ad libitum. All animal procedures were approved by the Institutional Animal Care and Use Committee (IACUC) at Columbia University Medical Center (Protocol # AC-AABB3552).

Note that full information on the approval of the study protocol must also be provided in the manuscript.
