# Supplementary figures and images for: The TINCR ubiquitin-like microprotein is a tumor suppressor in squamous cell carcinoma
Source: Nat Commun. 2023 Mar 10;14:1328. doi: 10.1038/s41467-023-36713-8 (PMC10006087; doi:10.1038/s41467-023-36713-8)

Figure 2, Panel a

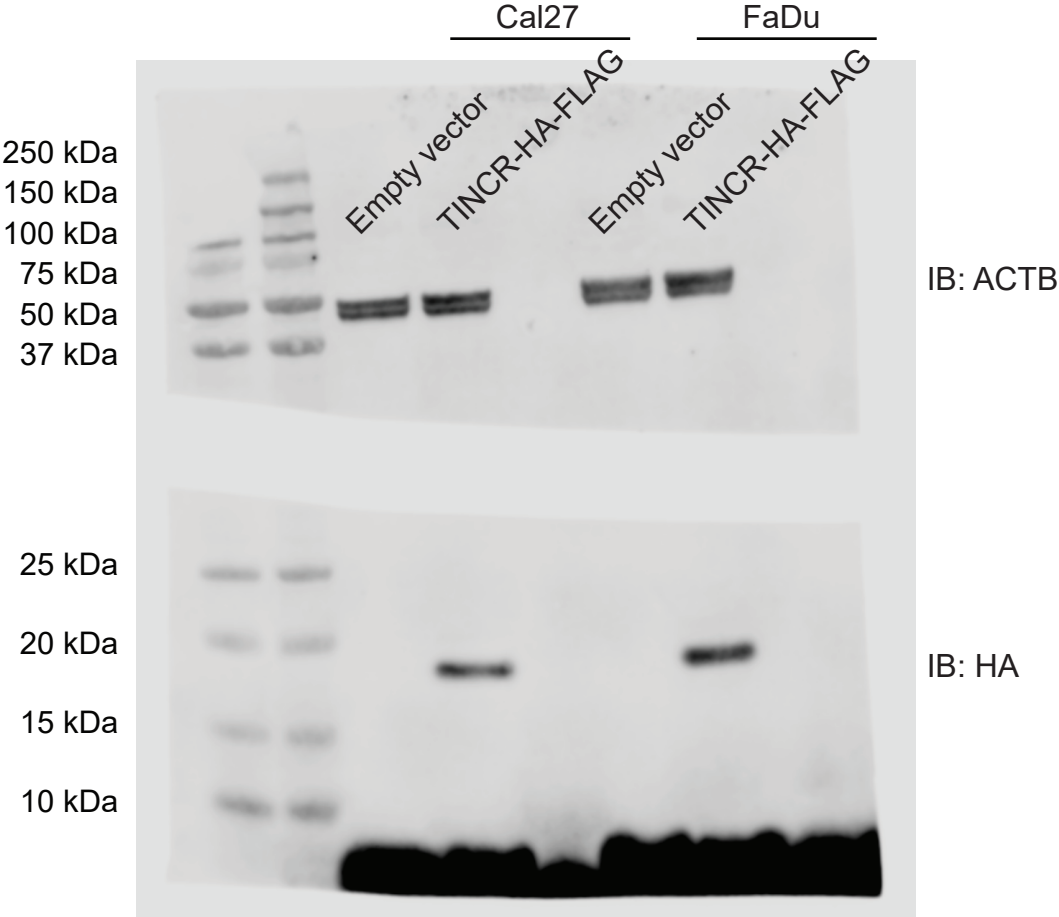

Figure 3, Panel h

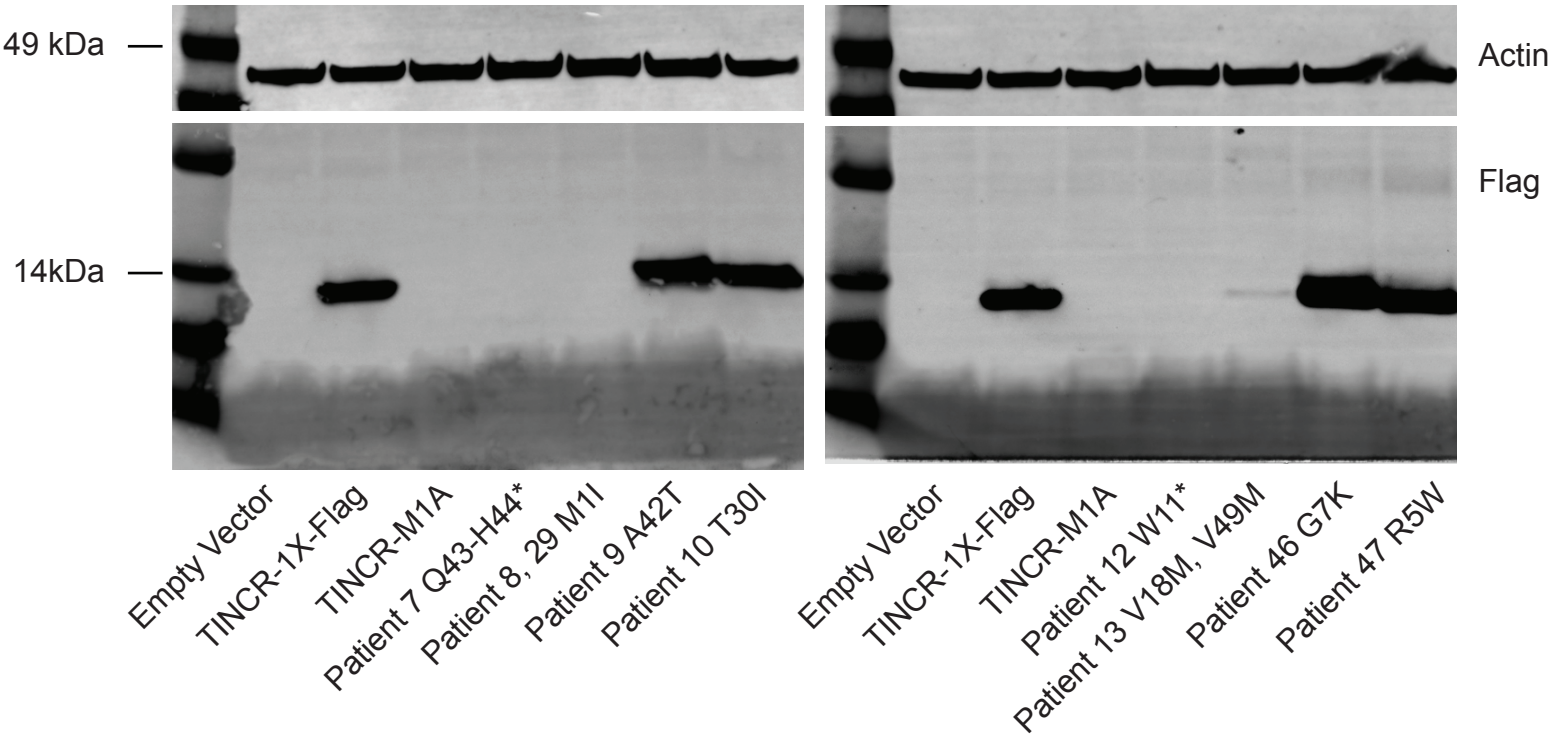

Supplemental Figure 1, Panel e

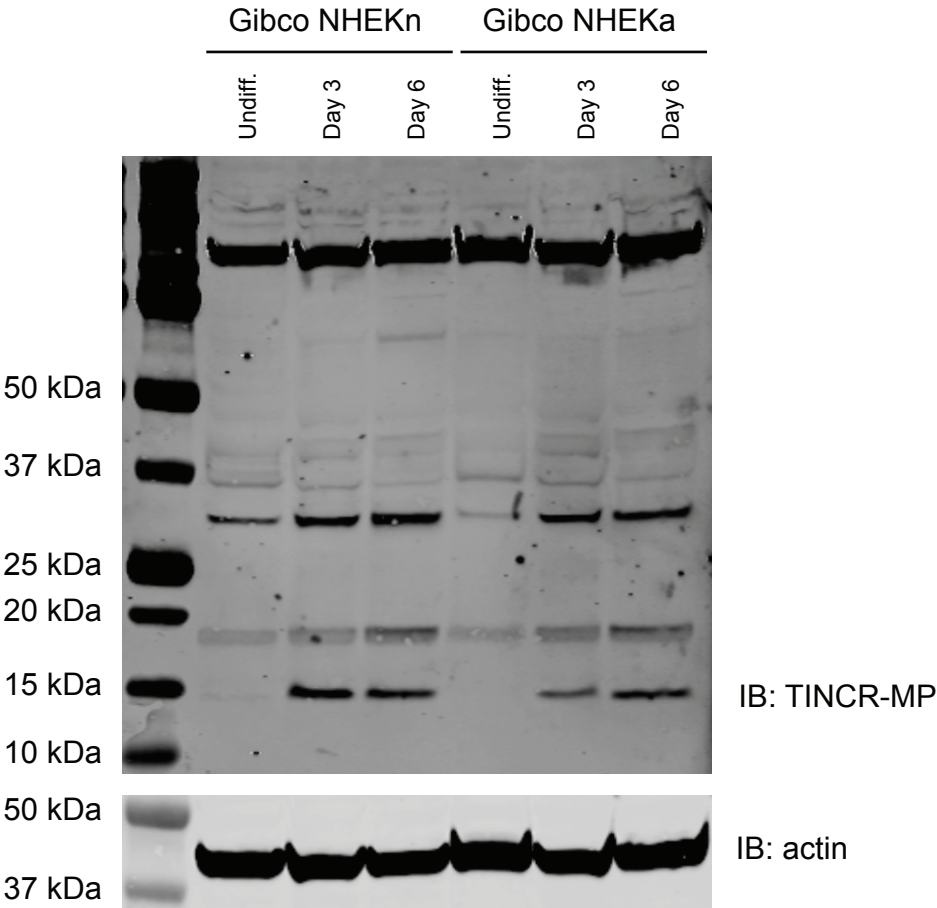

Supplementary Figure 3, Panel e

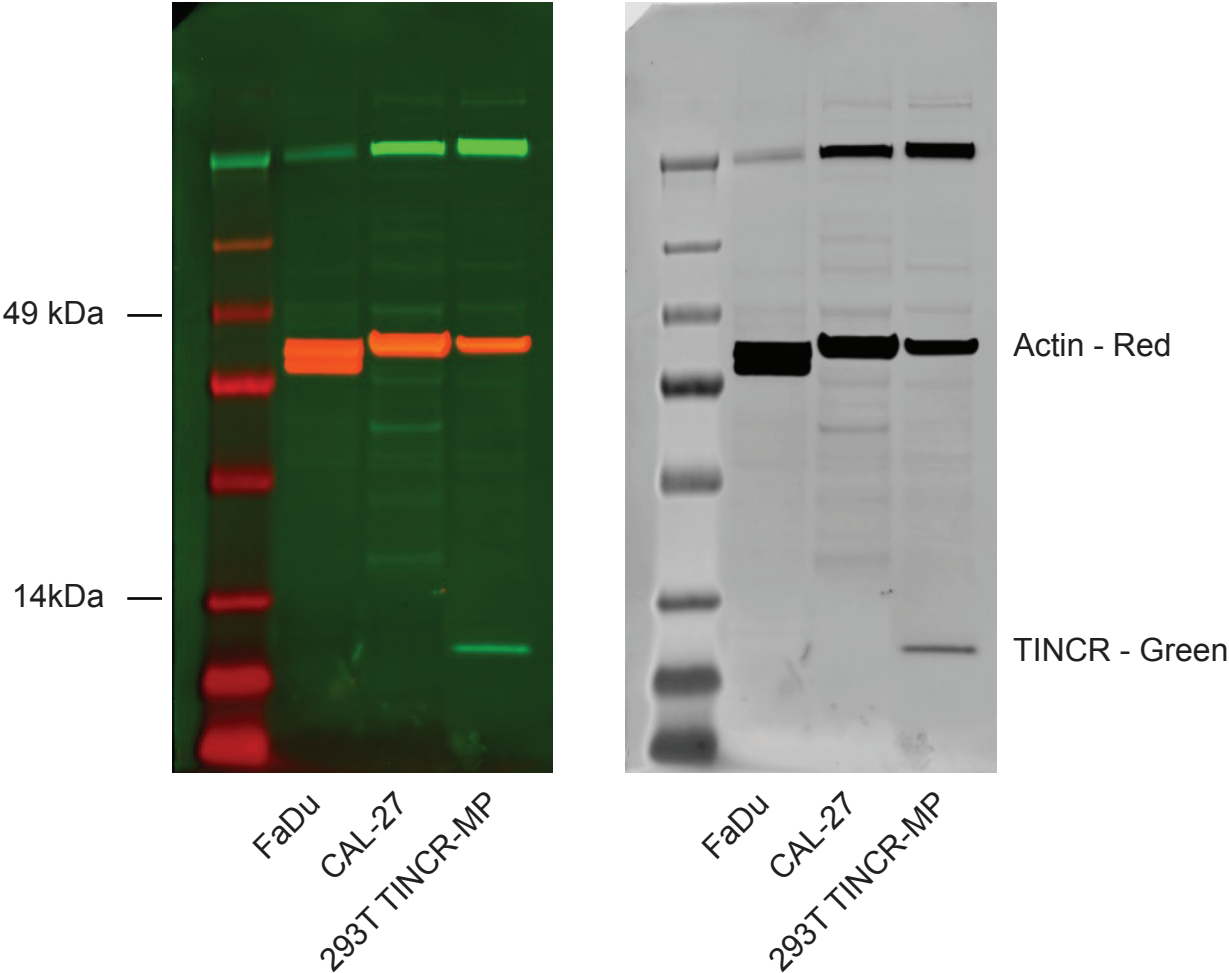

Supplement: Supplementary file 15 — Source Data [file 41467_2023_36713_MOESM15_ESM.zip › UncroppedWestern.pdf]
